# Multiomics insights into BMI-related intratumoral microbiota in gastric cancer

**DOI:** 10.3389/fcimb.2025.1511900

**Published:** 2025-02-18

**Authors:** Kang Liu, Zhengchen Jiang, Yubo Ma, Ruihong Xia, Yingsong Zheng, Kailai Yin, Chuhong Pang, Li Yuan, Xiangdong Cheng, Zhuo Liu, Bo Zhang, Shi Wang

**Affiliations:** ^1^ The Second Clinical Medical College of Zhejiang Chinese Medical University, Hangzhou, Zhejiang, China; ^2^ Department of Gastric Surgery, Zhejiang Cancer Hospital, Hangzhou Institute of Medicine (HIM), Chinese Academy of Sciences, Hangzhou, Zhejiang, China; ^3^ Zhejiang Key Lab of Prevention, Diagnosis and Therapy of Upper Gastrointestinal Cancer, Zhejiang Cancer Hospital, Hangzhou, Zhejiang, China; ^4^ Postgraduate training base Alliance of Wenzhou Medical University (Zhejiang Cancer Hospital), Hangzhou, Zhejiang, China; ^5^ Department of Integrated Chinese and Western Medicine, Zhejiang Cancer Hospital, Hangzhou Institute of Medicine (HIM), Chinese Academy of Sciences, Hangzhou, Zhejiang, China; ^6^ Zhejiang Provincial Research Center for Upper Gastrointestinal Tract Cancer, Zhejiang Cancer Hospital, Hangzhou, Zhejiang, China; ^7^ Endoscopy Division, Zhejiang Cancer Hospital, Hangzhou Institute of Medicine (HIM), Chinese Academy of Sciences, Hangzhou, Zhejiang, China

**Keywords:** GC, BMI, intratumoral microbiota, immune cells, metabolome

## Abstract

**Introduction:**

Body mass index (BMI) is considered an important factor in tumor prognosis, but its role in gastric cancer (GC) remains controversial. There is a lack of studies exploring the effect of BMI on gastric cancer from the perspective of intratumoral microbiota. This study aimed to compare and analyze the differences in and functions of intratumoral microbiota among GC patients with varying BMIs, aiming to ascertain whether specific microbial features are associated with prognosis in low-BMI (LBMI) gastric cancer patients.

**Methods:**

A retrospective analysis of the clinicopathological features and prognosis of 5567 patients with different BMIs was performed between January 2010 and December 2019. Tumor tissues from 189 GC patients were collected for 16S rRNA sequencing, 64 samples were selected for transcriptome sequencing, and 57 samples were selected for untargeted metabolomic analysis.

**Results:**

Clinical cohort analysis revealed that GC patients with a low BMI presented poorer clinical and pathological characteristics than those with a non-low-BMI (NLBMI). LBMI was identified as a significant independent risk factor for adverse prognosis, potentially exerting immunosuppressive effects on postoperative adjuvant chemotherapy. 16S rRNA sequencing revealed no significant differences in the alpha and beta diversity of the intratumoral microbiota between the two groups of GC patients. However, LEfSe analysis revealed 32 differential intratumoral microbiota between the LBMI and NLBMI groups. Notably, the genus Abiotrophia was significantly enriched in the LBMI group. Further in-depth analysis indicated that the genus Abiotrophia was inversely associated with eosinophils, P2RY12, and SCN4B genes, and positively linked with LGR6 in LBMI gastric cancer patients. Metabolomic assessments revealed that LBMI was positively associated with purine metabolites, specifically guanine and inosine diphosphate (IDP).

**Discussion:**

In conclusion, LBMI is an independent risk factor for poor prognosis in gastric cancer patients and may have an inhibitory effect on postoperative adjuvant chemotherapy. Intratumor flora of gastric cancer patients with different BMI levels differed, with different immune cell infiltration and metabolic characteristics. The genus Abiotrophia may promote gastric cancer development and progression by regulating eosinophils and the purine metabolism pathway, which provides a new idea for the precise treatment of gastric cancer.

## Introduction

Gastric cancer (GC) is the fifth most common malignancy globally and the fifth leading cause of cancer-related deaths (Bray et al., 2024). Over the past two decades, the 5-year survival rate of patients with GC has significantly improved due to various factors such as early detection, improvement in surgical techniques, improvement in nutritional care, and widespread use of systemic chemotherapy and immune-targeted therapy (Ahn et al., 2011). However, in China, most GC patients are diagnosed at an advanced or even late stage, with a higher proportion of patients experiencing significant weight loss and worse prognosis (Li et al., 2022).

BMI is a measure of body weight. It is an important prognostic factor for various tumors, such as colorectal cancer, breast cancer, and pancreatic cancer (Chen et al., 2024). However, its role in regulating the prognosis of patients with tumors including those with GC, is still controversial (Schooling et al., 2015; Feng et al., 2018; Ma et al., 2021; Zhao et al., 2021). Ma et al. (2021) demonstrated that GC patients with LBMI had a poor long-term prognosis, while Feng et al. (2018) found that GC patients with a high BMI had a better long-term prognosis. Interestingly, Schooling et al. (2015) found that obese patients had a high risk of death and poor prognosis. However, Zhao et al. (2021) showed no association between BMI and GC prognosis.

Previous studies have shown that intratumoral microbiota may contribute to tumorigenesis and progression and impact prognosis by inducing genomic instability and mutations affecting epigenetic modifications, promoting inflammatory responses, averting immune destruction, regulating metabolism, and activating invasion and metastasis (Wang et al., 2023; Cao et al., 2024; Liu et al., 2024). For example, *Fusobacterium nucleatum* is more abundant in various tumors such as colorectal cancer(CRC), oral cancer, and gastric cancers and affects long-term prognosis (Mitsuhashi et al., 2015; Mima et al., 2016; Hsieh et al., 2022), A novel virulence protein of *Fusobacterium nucleatum*, Fn-Dps, has been found to promote invasion and metastasis of CRC cells by inducing EMT through upregulation of the chemokine CCL2/CCL7 (Mima et al., 2016). Interestingly, two recent studies have demonstrated the heterogeneity of microorganisms at different BMI states (Huang et al., 2024; Li et al., 2024). In one of them, Huang et al (Huang et al., 2024). Similarly, in their study of CRC patients with different BMIs states similarly found the same significant enrichment at the portal level was detected in hyper-reorganized CRC patients, with significant enrichment of *Actinobacteria* spp*,Desulfovibrio* spp, and *Mycobacterium* spp at the genus level. Another study found that Peptostreptococcus stomatis was elevated in obese patients and that there were differential changes in metabolites between the two BMI groups, particularly in fatty acid and phospholipid dysregulation (Li et al., 2024). A study on intratumoral microbiota and GC revealed that *Methylobacterium tumefaciens* was significantly associated with poor prognosis in gastric cancer patients and was negatively correlated with CD8+ tissue-resident memory T (TRM) cells and TGF-β in the tumor immune microenvironment (TIME).Experimental methods verified that *Methylobacterium* could reduce TGF-β expression and the number of CD8^+^ TRM cells in tumors. These findings suggest that intratumoral microbiota may regulate the development of GC by influencing the tumor immune microenvironment (Peng et al., 2022).

Therefore, intratumoral microbiota have attracted increasing attention as influencing factors of the TIME. However, few studies have been conducted on GC, especially on LBMI GC patients with associated immunosuppression or intolerance (Indini et al., 2021). Therefore, in this study, we performed a multiomics analysis based on intratumoral microbiotas combined with transcriptomics and metabolomics to analyze intratumoral microbes and their functions in GC patients with different BMIs to understand the characteristics of the differential intratumoral microbes of LBMI GC patients, i.e., the mechanism of potential modulation of GC, and to provide a new solution for the precision treatment of GC.

## Material and methods

### Clinical cohort data collection and definitions

A retrospective analysis was conducted on 7,192 patients who underwent gastrectomy at Zhejiang Cancer Hospital from January 2010 to December 2019. Among them, 5,567 patients met the following inclusion criteria: 1.Preoperative pathological biopsy confirmed primary gastric cancer; 2.Underwent radical or palliative gastrectomy; 3.No concomitant severe diseases such as acute cardiovascular and cerebrovascular diseases, liver cirrhosis, and chronic renal failure. Exclusion criteria: 1.Received neoadjuvant treatments such as preoperative radiotherapy, chemotherapy, or immunotherapy; 2.Number of dissected lymph nodes<16; 3.Presence of other heterogeneous tumors; 4.Other types of gastric cancer (e.g.,neuroendocrine carcinoma, squamous cell carcinoma, adenosquamous carcinoma); 5.Patients with missing critical clinical data. The median follow-up time was 85 months (interquartile range:71 months). All eligible patients underwent radical gastrectomy according to the Japanese gastric cancer treatment guidelines (Association JGC, 2020). Surgical methods included proximal, total, and distal gastrectomy. Postoperatively, specimens were reviewed by pathology experts at the Cancer Hospital of the Chinese Academy of Sciences. Pathological tumor-lymph node metastasis (pTNM) staging was based on the 8th edition of the American Joint Committee on Cancer (AJCC) TNM staging system (Amin et al., 2017). Potential curative resection was defined as R0 resection. Survival time was calculated from the date of surgery to the date of GC-related death or the most recent follow-up. The follow-up cut-off date was August 1, 2023. Perioperative management followed routine procedures, with no differences between groups. Patients meeting the above criteria were divided into two groups according to the Preoperative BMI standards set by WHO: the low BMI group (BMI<18.5 kg/m²) and the non-low BMI group (BMI≥18.5 kg/m²). Various clinicopathological characteristics, surgery-related indicators, and postoperative outcome factors were collected for analysis, including gender, height, and weight. BMI was calculated based on the patients’ height and weight. Tumor location was classified according to the center of the lesion as Upper 1/3 (cardia, fundus), Middle 1/3 (body), Lower 1/3 (antrum, including the angular incisure and pylorus), or involving the entire stomach (Total) (tumor involving more than 2/3 of the stomach wall). Tumor size was determined by the maximum diameter of the tumor. The positive levels of tumor markers were defined as CA199 ≥ 37 U/ml and CEA ≥ 5 ng/ml.

### Clinical specimen collection and preparation

Samples were collected from 335 patients between January 2013 and December 2018 from Zhejiang Cancer Hospital. After screening according to the above clinical cohort criteria and ensuring that no antibiotics or intestinal microecological agents had been used in the previous month, 198 eligible GC patients were included in the final analysis. All patients were followed up by telephone and outpatient clinics with a follow-up cut-off date of 1 August 2023.The study was conducted by the Zhejiang Cancer Hospital (ZCH). The study was approved by the Ethics Committee of Zhejiang Cancer Hospital (approval number: IRB-2023-791) and written informed consent was obtained from all participants. Gastric samples were collected from patients who underwent gastrectomy, with peritumoral tissue 2-5 cm from the tumor margin. Notably, for metabolomics analysis, tissue specimens were subjected to cold ischemia for less than 30 minutes before freezing at -80 degrees Celsius. For 16S rRNA sequencing and transcriptome analyses, tissue specimens were immersed in an RNA-protecting solution at 4°C overnight, and then frozen at -80°C. Specimens for each histology were collected simultaneously. All tissue samples were collected at the time of surgical specimen removal. Histological sections at the top and bottom of each specimen were reviewed by a senior board-certified pathologist to confirm whether the tissue was tumor tissue or adjacent non-tumor tissue. For the purposes of this study, tumor samples had to have an average of 60% tumor cell nuclei and less than 20% necrosis to qualify.

### 16S rRNA sequencing

Microbial DNA was extracted using an E.Z.N.A. Tissue DNA Kit (D3396-01; Omega, Norcross, Georgia, USA) following the manufacturer’s instructions as described previously. The DNAs were quantified using a Qubit 2.0 Fluorometer (Invitrogen, Carlsbad, CA, USA), and molecular size was estimated using agarose gel electrophoresis. Primers targeting the hypervariable V3-V4 region of the 16S rRNA gene were used to amplify the extracted DNA samples. The forward primer was 5’-CCTACGGGNGGCWGCAG-3’ and the reverse primer was 5’-GACTACHVGGGTATCTAATCC-3’. AxyPrep PCR Clean-up Kit (AP-PCR-500G; Corning, NY, USA) was used to separate, extract and purify the PCR products, and the products were quantified using a Quant-iT PicoGreen dsDNA Reagent (P7581, Thermo Scientific, Waltham, MA, USA). After quality determination, libraries passing quality control were sequenced with Novaseq sequencer for 2 x Two terminal sequencing of 250 bp at LC-Bio Co., Ltd.

Species annotation of the colonies was performed using the Greengene database v13.8, and then the ASV/OUT data of the colonies were extracted using the phyloseq package v1.26.1. We used the α-diversity index to characterize the diversity of the flora, where Shannon and Simpson indices were used to characterize species richness, homogeneity, and concentration reflecting species diversity, respectively. Beta diversity was calculated based on weighted Unifrac distances, and principal coordinate analysis (PCoA) was used in order to assess differences in microbial community composition. Linear discriminant analysis (LDA) was performed using the Mann-Whitney U test, and linear discriminant analysis effect size (LEfSe) analysis was performed using lefse software v1.0.0 to screen for species most likely to explain differences between groups, while LDA scores were used to assess effect sizes for species with significant differences between groups, with |LDA |> 2 and P < 0.05 as the thresholds of difference to screen for differences between species, and ggplot 2 software was used to assess differences in the composition of microbial communities. The results were also analyzed as bar graphs using the ggplot 2 software package v3.4.0. The results were presented as bar graphs. The α-diversity, β-diversity indices between the two groups were compared using Mann-Whitney U rank sum test through vegan software package v2.5.6. All the above analyses were carried out in R software v4.3.1, and the above P-values were two-tailed tests, and differences were considered statistically significant when P<0.05.

### Transcriptome sequencing

Paired tumor tissues from 108 GCs were subjected to mRNA sequencing (RNA-seq).In the end, 64 samples met the screening criteria. Total RNA was isolated from tumor tissues and NATs using TRIzol reagent (Invitrogen, Carlsbad, CA, USA) in an RNA protection solution. the amount and purity of RNA from each sample was quantified using a NanoDrop ND-1000 (NanoDrop, Wilmington, DE, USA). RNA integrity was assessed using an Agilent 2100 with a RIN>7.0. For mRNA sequencing, libraries were prepared on 1 μg of DNase I-treated total RNA using the TruSeq kit (Illumina) and processed for 150 bpb on the Illumina HiSeq X Ten instrument at LC-Bio Technology Co. (Hangzhou, China) on an Illumina HiSeq X Ten instrument with 150-bp paired-end sequencing. (Hangzhou, China) performed 150-bp paired-end sequencing on an Illumina HiSeq X Ten machine according to the protocol recommended by the vendor.

We aligned reads of all samples to the < research species > reference genome using HISAT2 (https://daehwankimlab.github.io/hisat2/, version:hisat2-2.0.4) package, which initially remove a portion of the reads based on quality information accompanying each read and then maps the reads to the reference genome. HISAT2 allows multiple alignments per read (up to 20 by default) and a maximum of two mismatch when mapping the reads to the reference. HISAT2 build a database of potential splice junctions and confirms these by comparing the previously unmapped reads against the database of putative junctions. The mapped reads of each sample were assembled using StringTie (http://ccb.jhu.edu/software/stringtie/, version:stringtie- 1.3.4d) with default parameters. Then, all transcriptomes from all samples were merged to reconstruct a comprehensive transcriptome using gffcompare software (http://ccb.jhu.edu/software/stringtie/gffcompare.shtml, version: gffcompare-0.9.8). After the final transcriptome was generated, StringTie and ballgown (http://www.bioconductor.org/packages/release/bioc/html/ballgown.html) were used to estimate the expression levels of all transcripts and perform expression abundance for mRNAs by calculating FPKM (fragment per kilobase of transcript per million mapped reads) value.

Differentially expressed genes (DEGs) were screened by DESeq261. genes with P< 0.05 and FC ≥ 2 or FC ≤ 0.5 were considered statistically significant DEGs. enriched functional pathways and modules were analyzed by using KEGG and CO databases. The Mann-Whitney U test was used to compare differences between groups.

### GC tumor immune microenvironment analysis

CIBERSORT is a computational method for analyzing the composition of immune cells from RNA sequencing data based on the expression profiles of immune cell-specific genes and uses machine learning algorithms to analyze and classify the expression patterns of these genes. We use the CIBERSORT R-script v1.03 to construct a support vector regression-based model using the known expression data of the reference genes and to-be-estimated gene expression data of the mixed samples, constructed the optimization problem by the correlation matrix consisting of the cellular composition, and solved it in the form of a sparse solution. Thus, the cellular composition ratio of the mixed samples is estimated. The FPKM matrix obtained by transcriptome sequencing was transformed into a matrix of relative content of 22 different types and functional states of immune cells. The flora matrix was combined with the immune cell abundance matrix and the correlation coefficients between the columns in the combined matrix were calculated by calling the rcorr function. The type of correlation coefficient was Spearman’s correlation coefficient.

### Metabolome assays

The samples were taken out of the -80°C freezer and thawed on ice, and metabolite were extracted with 80% methanol buffer. Briefly, 50 mg of sample was extracted with 0.5 ml of precooled 80% methanol. The extraction mixture was then stored in 30 min at -20°C. After centrifugation at 20,000 g for 15 min, the supernatants were transferred into new tube to and vacuum dried. The samples were redissolved with 100 μL 80% methanol and stored at -80°C prior to the LC-MS analysis. In addition, pooled QC samples were also prepared by combining 10 μL of each extraction mixture. The extracted samples were then sorted for machine analysis with randomization. QC samples were inserted before, in the middle, and after the samples to evaluate experimental technical replicates. The samples underwent mass spectrometry positive and negative ion scans. All samples were acquired by the LC-MS system followed machine orders. Firstly, all chromatographic separations were performed using an UltiMate 3000 UPLC System (Thermo Fisher Scientific, Bremen, Germany). An ACQUITY UPLC T3 column (100mm×2.1mm,1.8μm, Waters, Milford, USA) was used for the reversed phase separation. The column oven was maintained at 40°C. A high-resolution tandem mass spectrometer TripleTOF 6600 (SCIEX, Framingham, MA, USA) was used to detect metabolites eluted form the column. The Q-TOF was operated in both positive and negative ion modes. The curtain gas was set 30 PSl,lon source gas1 was set 60 PSI,lon source gas2 was set 60 PSI, and an interface heater temperature was 500°C. For positive ion mode, the lonspray voltage floating were set at 5000 V, respectively. For negative ion mode, the lonspray voltage floating were set at -4500V, respectively. The mass spectrometry data were acquired in IDA mode. The TOF mass range was from 60 to 1200 Da. The survey scans were acquired in 150 ms and as many as 12 production scans were collected if exceeding a threshold of 100 counts per second (counts/s) and with a 1^+^charge-state. Dynamic exclusion was set for 4s. During the acquisition, the mass accuracy was calibrated every 20 samples. Furthermore, in order to evaluate the stability of the LC-MS during the whole acquisition, a quality control sample (Pool of all samples) was acquired after every 10 samples.

The raw data from mass spectrometry were converted into readable data mzXML format using Proteowizard’s MSConvert software. XCMS software was utilized for peak extraction, and peak extraction quality control was conducted. Subsequently, substances extracted were annotated using CAMERA for adduct and ion annotation, followed by primary identification using the metaX software. Identification was performed separately using the mass spectrometry first-level information and matching the mass spectrometry second-level information with an in-house standard compound database. Differential metabolites were identified by Mann-Whitney U test and partial least squares discriminant analysis (PLS-DA).Metabolites with variable importance in projection (VIP) > 1 and p < 0.05 and FC ≥ 2 or FC ≤ 0.5 were considered differential metabolites. The functions of these metabolites and metabolic pathways were analyzed using the KEGG database.

### Statistical method

Continuous variables with normal distribution are expressed as mean ± standard deviation (x ± s) or Mean ± SD and analyzed using t-test or Mann-Whitney U test. Categorical variables are presented as counts (n, %) and analyzed using Chi-square test or Fisher’s exact test. Propensity score matching (PSM) was used to account for differences in patient backgrounds, with a 1:4 ratio set to minimize selection bias between the two groups. Survival rates were calculated using the Kaplan-Meier method and survival curves were compared using the log-rank test. A Cox proportional hazards model with forward stepwise regression was employed to identify independent prognostic factors. Spearman correlation was used for the joint analysis of microbiome with transcriptome and metabolome. All data were analyzed using SPSS software version 26.0 (IBM USA), the Medsta statistical platform (www.medsta.cn/software), OmicStudio tools (https://www.omicstudio.cn/tool), and R version 4.3.1. All statistical tests were two-sided, and a p-value < 0.05 was considered statistically significant.

## Results

### LBMI is an independent prognostic risk factor for poor prognosis in patients with GC

**Figure 1:**
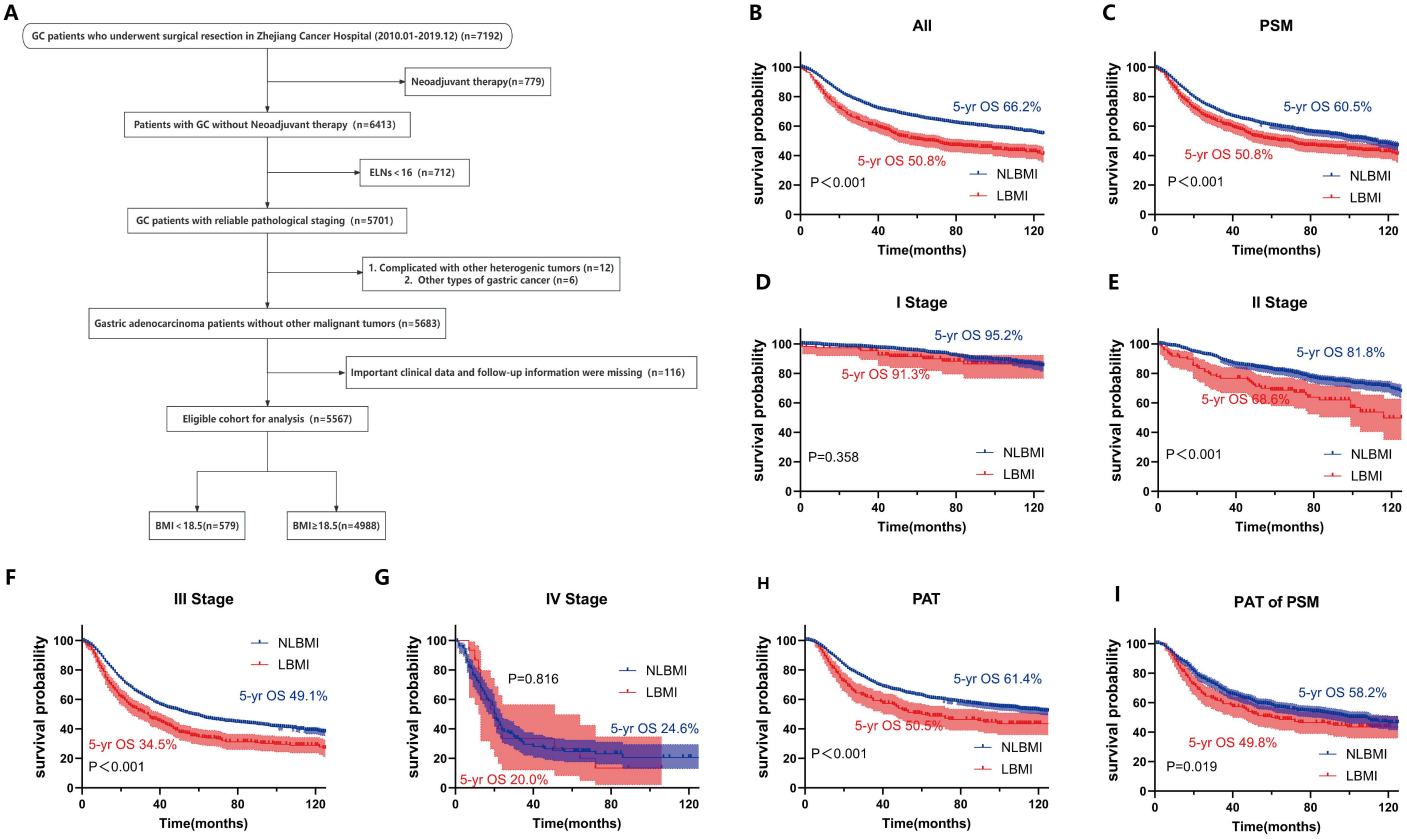
LBMI is an independent risk factor for poor prognosis in GC. **(A)** Clinical cohort screening flowchart. Kaplan-Meier survival curve analysis for different cohorts classified by BMI. **(B)** All patients. **(C)** All matched patients. **(D)** Stage I **(E)** Stage II. **(F)** Stage III. **(G)** Stage IV. **(H)** PAT. **(I)** PAT of PSM. BMI, Body Mass Index. ELNs, Number of dissected lymph nodes. PSM, Propensity Score Matching. PAT, Postoperative Adjuvant Therapy. LBMI, Low Body Mass Index (BMI < 18.5). NLBMI, Non-Low Body Mass Index (BMI ≥ 18.5).All P values for survival curves were corrected for multiplicity by the BH method.

In this study, data from 5567 patients who met the criteria and had complete follow-up information were collected from 7192 hospitalized patients with GC (**Figure 1A**). There were no statistically significant differences between the two groups of BMI patients in terms of smoking history, alcohol consumption history, extent of resection, type of pathology, pM stage or recurrent metastasis (all P > 0.05). Analysis revealed that relative to NLBMI patients, LBMI patients had a smaller percentage of family history of GC; more tumors were located in the lower 1/3 and the whole stomach and less in the upper 1/3, and there was a greater percentage of larger and more poorly differentiated tumors, and a greater percentage of open surgeries (all P < 0.05); the level of pre-CA199 positivity was significantly greater (P=0.039), and the pre-CEA positivity level was similar; and the percentage of nerve invasion was greater (P=0.011), while there was no significant difference in vascular invasion. Moreover, in the LBMI group, the percentage of female patients aged ≥60 years, incidence of complications, deep tumor infiltration, high number of lymph node metastases, late pathological stage and low percentage of receiving postoperative adjuvant chemotherapy were significantly greater than that those in the NLBMI group (P<0.001). (**Supplementary Table S1**).

Univariate and multivariate COX analysis revealed that LBMI is an independent poor prognostic factor for overall survival (OS) in GC patients (HR=1.28, 95%CI: 1.13-1.45, P<0.001) (**Table 1**). Kaplan-Meier survival analysis based on BMI classification showed that LBMI patients had worse prognosis compared to NLBMI patients before PSM (5-yr OS: 50.8% vs. 66.2%, P<0.001) (**Figure 1B**). After adjusting for clinicopathological characteristics that influence prognosis (P<0.05) using PSM (ratio 1:4), the clinical characteristics of the two groups were comparable (P>0.05, **Supplementary Table S2**). Similarly, LBMI patients had worse prognosis (5-yr OS: 50.8% vs. 60.5%, P<0.001) (**Figure 1C**). Stratified analysis by TNM stage show no significant difference in OS between the two BMI groups in stage I and IV disease (**Figures 1D, G**); however, in stage II and III patients, LBMI disease have worse OS compared to NLBMI patients (**Figures 1E, F**). Subgroup analysis based on receiving postoperative chemotherapy set the PSM ratio to 1:4, and included clinicopathological data that influence prognosis. After PSM, the clinicopathological characteristics of the two BMI groups were comparable (**Supplementary Table S3**), and LBMI patients had worse OS both before and after PSM (**Figures 1H, I**).

**Table 1:** Single factor and multi-factors Cox analysis risk factor for gastric cancer OS.

Parameters	Univariate	P value	Multivariate	P value
	HR (95%CI)		HR (95%CI)	
Gender(Male vs Female)	1.21 (1.10 ~ 1.34)	<0.001		
Age (≥60years vs<60years)	1.65 (1.51 ~ 1.80)	<0.001	1.37 (1.25 ~ 1.51)	<0.001
BMI(<18.5 vs ≥18.5)	1.60 (1.41 ~ 1.80)	<0.001	1.28 (1.13 ~ 1.45)	<0.001
Family.history	0.91 (0.83 ~ 0.99)	0.041		
Smoking.history	1.06 (0.97 ~ 1.15)	0.171		
Drinking.history	1.03 (0.94 ~ 1.13)	0.531		
Surgery.methods(Laparoscopy vs Open)	0.50 (0.43 - 0.58)	<0.001	0.78 (0.68 - 0.91)	<0.001
Tumor location		<0.001		0.002
Upper1/3	Ref		Ref	
Middle1/3	0.53 (0.46 ~ 0.61)	<0.001	0.88 (0.76 ~ 1.02)	0.097
Lower1/3	0.61 (0.55 ~ 0.67)	<0.001	0.90 (0.82 ~ 0.99)	0.039
Total	2.26 (1.84 ~ 2.77)	<0.001	1.46 (1.18 ~ 1.80)	<0.001
Pathological type		0.042		<0.001
Adenocarcinoma	Ref		Ref	
MGC	0.87 (0.66 ~ 1.13)	0.296	0.62 (0.47 ~ 0.81)	<0.001
SRCC	1.23 (1.05 ~ 1.43)	0.010	1.34 (1.14 ~ 1.57)	<0.001
Differentiation		<0.001		
Poorly	Ref			
Moderately	0.37 (0.23 ~ 0.59)	<0.001		
Well	0.79 (0.70 ~ 0.89)	<0.001		
Vascular.tumor.thrombus	2.45 (2.25 ~ 2.68)	<0.001	1.38 (1.26 ~ 1.52)	<0.001
Nerve.invasion	2.95 (2.68 ~ 3.24)	<0.001	1.42 (1.28 ~ 1.58)	<0.001
Maximum tumor diameter(≥5cm vs <5cm)	2.92 (2.68 ~ 3.18)	<0.001	1.57 (1.43 ~ 1.72)	<0.001
pTNM Satge		<0.001		<0.001
I	Ref		Ref	
II	3.14 (2.53 ~ 3.91)	<0.001	2.23 (1.77 ~ 2.79)	<0.001
III	9.31 (7.72 ~ 11.23)	<0.001	4.90 (3.95 ~ 6.07)	<0.001
IV	18.67 (14.43 ~ 24.15)	<0.001	10.59 (8.00 ~ 14.02)	<0.001
Pre-CEA	1.89 (1.72 ~ 2.08)	<0.001	1.31 (1.19 ~ 1.45)	<0.001
Pre-CA199	2.13 (1.93 ~ 2.34)	<0.001	1.26 (1.14 ~ 1.39)	<0.001
Postoperative adjuvant therapy	1.14 (1.05 ~ 1.24)	0.002	0.71 (0.65 ~ 0.78)	<0.001

BMI: Body Mass Index,MGC: Mucinous adenocarcinoma,SRCC:signet-ring cell carcinoma,Pre-:Pre-operation.P < 0.05 was considered significant. All P values were corrected by BH method.

### Intratumoral microbiome landscape in LBMI and NLBMI gastric cancer patients

**Figure 2:**
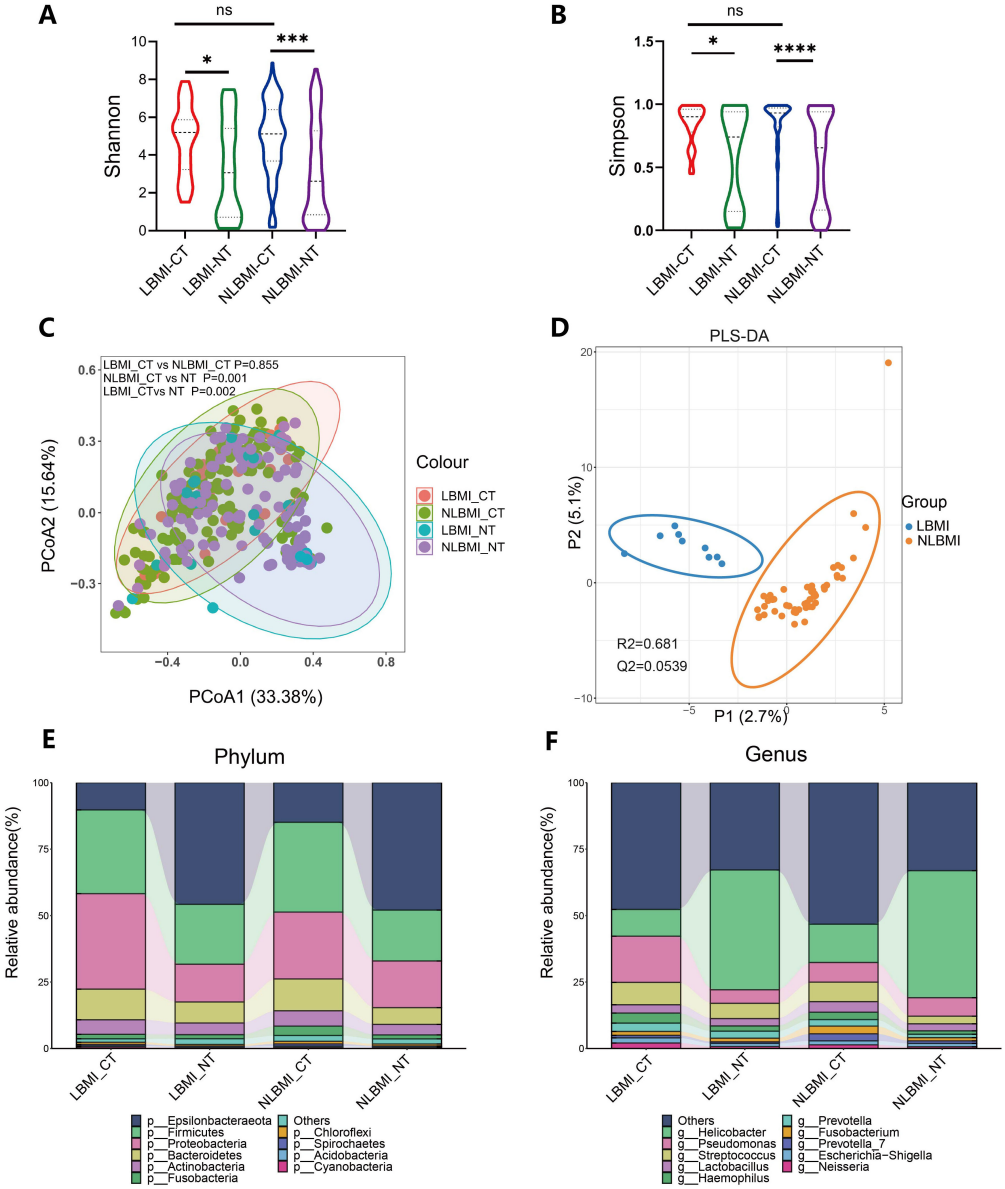
Tumor microbiome landscape of LBMI and NLBMI gastric cancer patients. Alpha diversity analysis of the LBMI and NLBMI groups. **(A)** Shannon index and **(B)** Simpson index in gastric cancer samples of each group. **(C)** Beta diversity analysis using UniFrac distance-weighted PCoA shows differences between cancerous and adjacent tissues in low BMI and non-low BMI groups. **(D)** PLS-DA analysis shows that the tumor microbiome composition of GC patients in the LBMI group and NLBMI group can be clearly divided into two different clusters. Stacked bar charts showing the species composition at **(E)** phylum level and **(F)** genus level for LBMI and NLBMI groups. LBMI-CT, Low BMI tumor tissue; LBMI-NT, Low BMI adjacent normal tissue; NLBMI-CT, Non-Low BMI tumor tissue; NLBMI-NT, Non-Low BMI adjacent normal tissue. PLS-DA, Partial Least Squares Discriminant Analysis. PCoA, Principal Coordinate Analysis. P < 0.05 is considered statistically significant. no * indicates P value ≥ 0.05, * indicates 0.01 ≤ P < 0.05, ** indicates 0.001 ≤ P < 0.01, *** indicates P < 0.001. ns, No sense.

To evaluate whether there were differences in microbial diversity, abundance, and composition between LBMI and NLBMI gastric cancer patients, we included 189 eligible gastric cancer patients, including 27 in the LBMI cohort and 162 in the NLBMI cohort (**Supplementary Figure S1**). As shown in **Supplementary Table S4**, the clinicopathologic data were balanced and comparable between the two cohorts. On the basis of the species sparsity curves (**Supplementary Figures S2B, C**), we found that the curves of the four groups in both metrics flattened out. The Venn diagram (**Supplementary Figure S2A**) revealed that there are many overlaps in the microbial environments among the four groups. The diversity within the cancer tissues was significantly greater in both LBMI and NLBMI carcinomas than in the LBMI and NLBMI paracarcinomas, whereas there was no significant difference between the two cohorts of LBMI and NLBMI cancer tissues (**Figures 2A, B**). Principal coordinate analysis (PCoA) revealed significant differences in both BMI carcinomas and paracancers in both groups; however, there was no significant difference between LBMI-CT and NLBMI-CT (P=0.855) (**Figure 2C**). PLS-DA analysis, revealed that the intratumoral microbiome of two groups of BMI carcinomas could be divided into two different clusters (**Figure 2D**). Regarding species composition, the differences were a smaller between the tumor tissues of different BMI groups, while the differences were a greater between tumor and peritumoral tissues of the same BMI group (**Figures 2E, F**).

### LBMI intratumor *g_Abiotrophia* was significantly elevated

**Figure 3:**
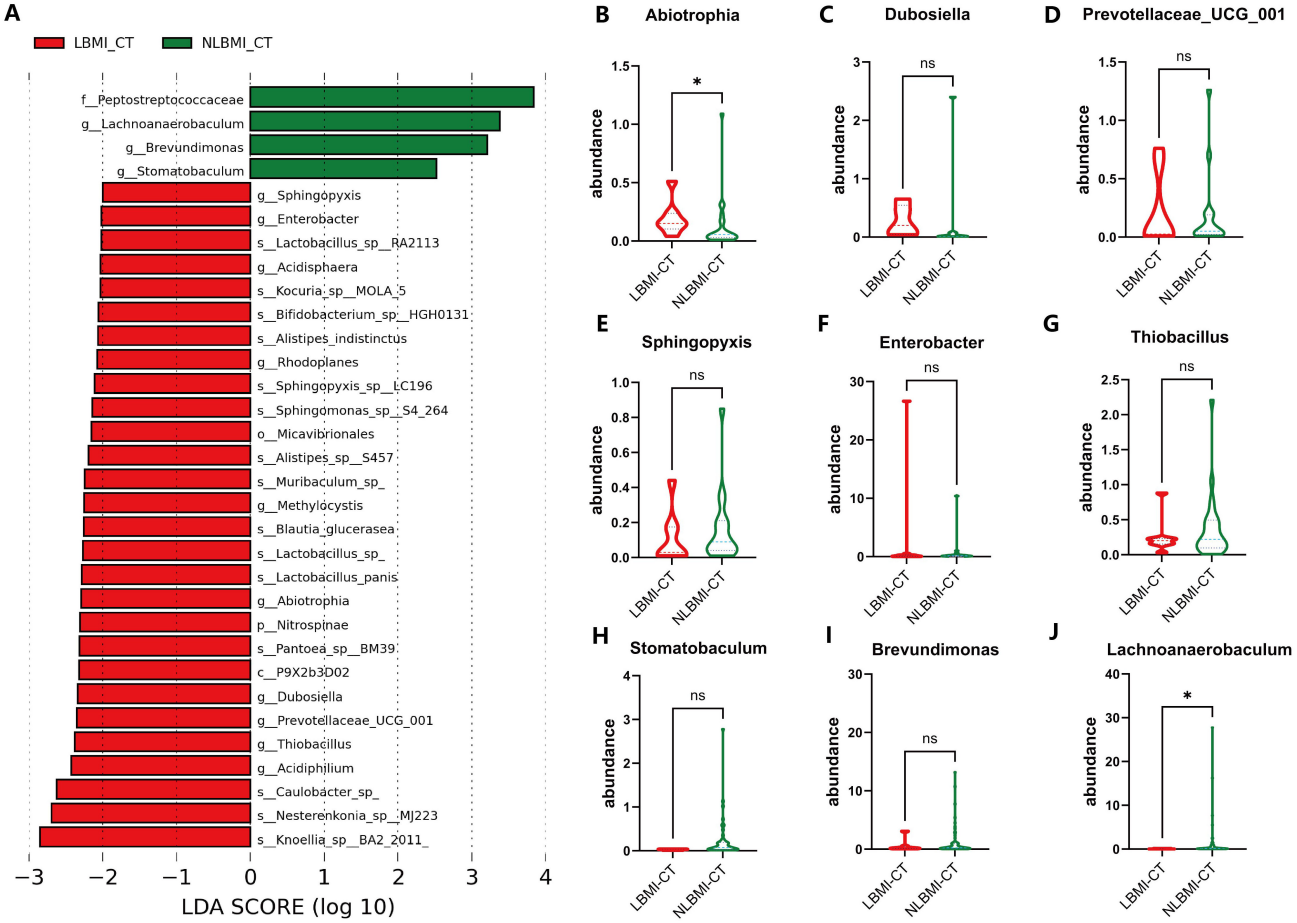
Significantly increased intratumoral *g_Abiotrophia* in LBMI. **(A)** Lefse analysis of LBM-CT and NLBMI-CT groups. The criterion for feature selection is an LDA score >2.0. The color of the bars represents the group, and the length of the bars represents the size of the LDA score. LDA score indicates the influence of the microbiota on LBMI and NLBMI groups. **(B-J)** Box plots of differential genus-level dominant bacteria abundance in GC patients between LBMI and NLBMI groups. P < 0.05 is considered statistically significant. The “*” in the figure indicates the significance level: no * indicates P value ≥ 0.05, * indicates 0.01 ≤ P < 0.05, ** indicates 0.001 ≤ P < 0.01, *** indicates P < 0.001. ns, No sense. LBMI-CT, Low BMI tumor tissue; NLBMI-CT, Non-Low BMI tumor tissue.

To determine the differentially dominant flora in GC patients with different BMIs, LEfSe analysis was performed (LDA> 2.0, P<0.05), which revealed 59 (**Supplementary Figure S3**) and 230 (**Supplementary Figure S4**) differentially dominant flora in the LBMI group and the NLBMI group, respectively, compared with the paracancerous tissues. There were 32 differentially dominant flora in the LBMI-CT group compared with the NLBMI-CT group. At the phylum level only *p_Nitrospinae* dominated the flora in LBMI, whereas at the genus level *g_Lachnoanaerobaculum,g_Brevundimonas* and *g_Stomatobaculum* dominated the flora in the NLBMI group, whereas *g_Acidiphilium,g_Thiobacillus* and *g_Abiotrophia* and 9 other genera were the dominant flora in the LBMI group. At the species level, *s_Knoelia_sp_BA2_2011* and 15 other species were dominant flora in the LBMI group (**Figure 3A**; **Supplementary Figure S5**). At the genus level, the abundances of two groups of differentially bacteria, *g_Abiotrophia* and *g_Lachnoanaerobaculum*, significantly differed (**Figures 3B-J**). Spearman correlation analysis revealed that g_Abiotrophia was positively correlated with *g_Lachnoanaerobaculum* and *g_Stomatobaculum* and that *g_Brevundimonas* was negatively correlated. These findings suggest a possible complementary relationship between the dominant differential flora between the two BMI groups (**Supplementary Figure S2D**).

### LBMI intratumoral *g_Abiotrophia* negatively correlates with P2RY12

**Figure 4:**
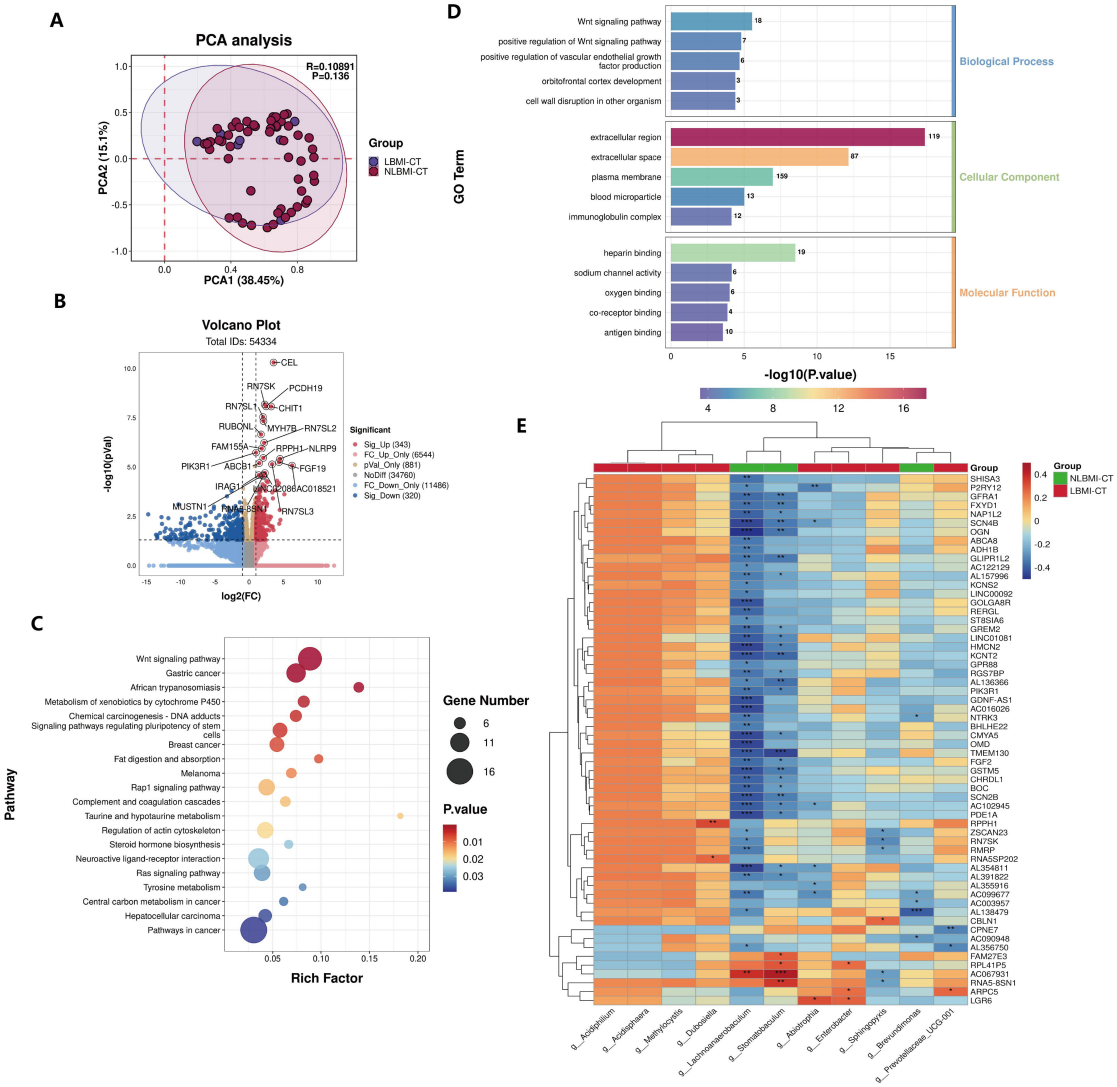
Negative correlation between intratumoral *g_Abiotrophia* and P2RY12 in LBMI. **(A)** Principal Component Analysis (PCA) of transcriptome samples from GC patients in the LBM-CT and NLBMI-CT groups. **(B)** Volcano plot of GC patients in the LBM-CT and NLBMI-CT groups, with selection criteria (|log2FC| ≥ 1, P < 0.05). **(C, D)** The function of these genes and transcription pathways was investigated using the KEGG and GO databases, and the TOP20 KEGG pathways were displayed in a bubble chart and the TOP15 GO pathways were shown in a bar chart. **(E)** Correlation heatmap showing the spearman analysis of the TOP60 differential genes and genus-level intratumoral bacteria. Red indicates positive correlation; blue indicates negative correlation. The color depth represents the magnitude of the correlation coefficient, with color ranging from light to dark indicating increasing correlation value. P < 0.05 is considered statistically significant. The “*” in the figure indicates the significance level: no * indicates P value ≥ 0.05, * indicates 0.01 ≤ P < 0.05, ** indicates 0.001 ≤ P < 0.01, *** indicates P < 0.001. LBMI-CT, Low BMI tumor tissue; NLBMI-CT, Non-Low BMI tumor tissue.

RNA sequencing analysis was performed on 64 tumor tissues from both groups, and PCA revealed that there was no significant difference in BMI the between the two groups (P=0.136) (**Figure 4A**). Compared with NLBMI, 343 genes were significantly upregulated and 320 genes in LBMI were significantly downregulated (**Figure 4B**). KEGG and GO analyses were performed on the BMI differential genes of the two groups, and KEGG analysis revealed that the LBMI group was enriched mainly in the Wnt signaling pathway, gastric cancer, and African trypanosomiasis (**Figure 4C**); similarly GO enrichment analysis was performed mainly in the extracellular region, extracellular space, plasma membrane and Wnt signaling pathway (**Figure 4D**). Correlation analysis of the DEGs associated with the dominant flora at the genus level revealed that *g_Abiotrophia* was significantly positively correlated with 11 genes, such as LGR6,and significantly negatively correlated with 30 genes, such as P2RY12 and SCN4B,in the LBMI group (**Figure 4E**; for details, see **Additional File S1**). The above results revealed that GC patients with different BMIs presented different transcriptomic landscapes and that many of these genes were closely related to differential intratumoral microbiota, suggesting that differential intratumoral microbiota may regulate the progression of GC by influencing the genes of the host.

### LBMI intratumoral *g_Abiotrophia* negatively correlates with eosinophils

**Figure 5:**
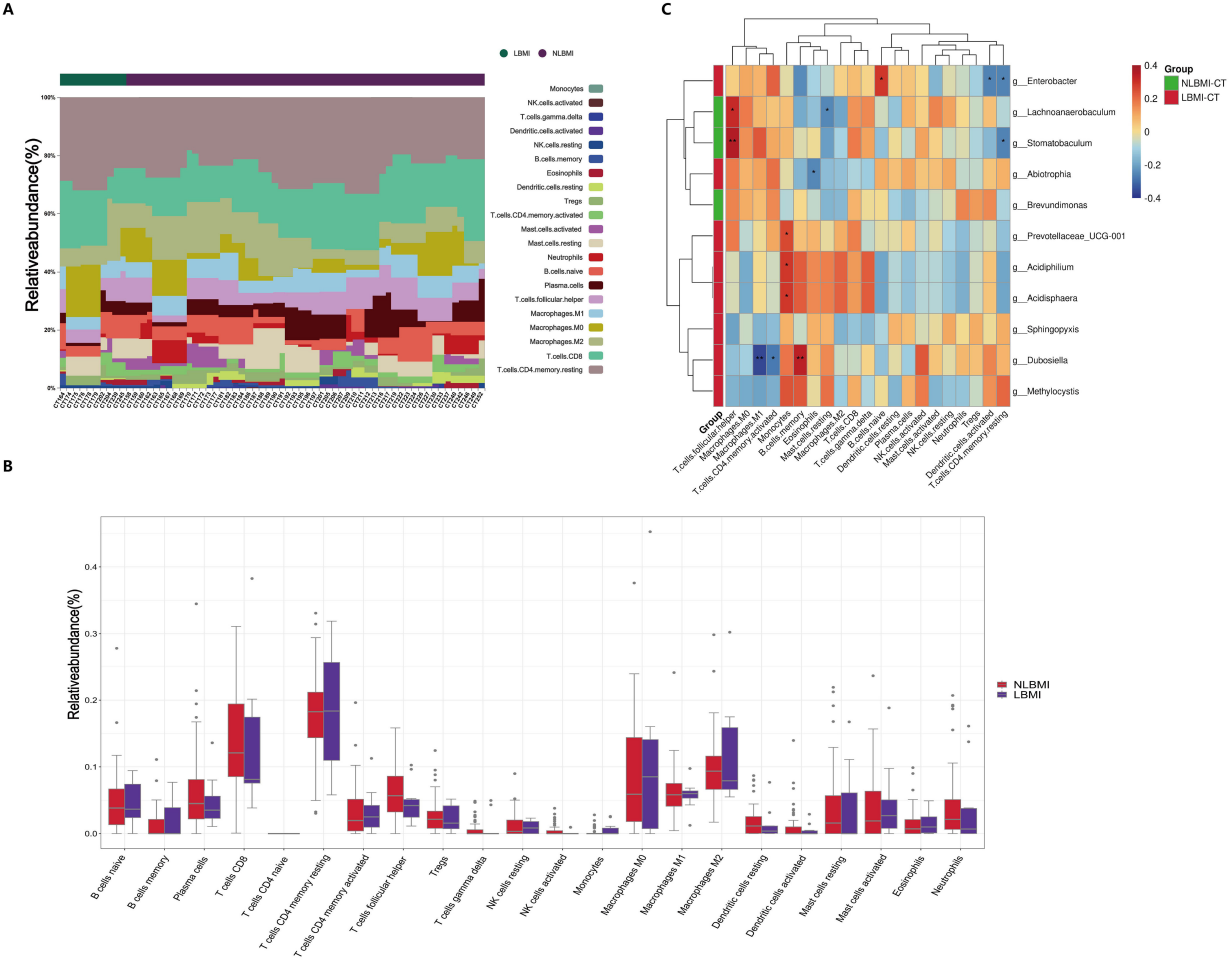
Negative correlation between intratumoral *g_Abiotrophia* and eosinophils in LBMI. **(A)** Bar chart of the relative abundance of 22 immune cells in GC patients grouped by BMI status. Each bar represents a sample, with each color corresponding to a different immune cell type. The y-axis represents the relative abundance values of the immune cells, with the sum of the relative abundance of all immune cells in a single sample equal to 1. **(B)** Box plot showing differences in the abundance of tumor-infiltrating immune cells between LBMI and NLBMI groups. **(C)** Correlation heatmap showing spearman analysis between tumor-infiltrating immune cells and genus-level intratumoral bacteria. The x-axis represents immune cells, and the y-axis represents bacteria. Red indicates positive correlation; blue indicates negative correlation. The color depth represents the magnitude of the spearman correlation coefficient, with color ranging from light to dark indicating increasing correlation value. The “*” in the figure indicates the significance level: no * indicates P value ≥ 0.05, * indicates 0.01 ≤ P < 0.05, ** indicates 0.001 ≤ P < 0.01, *** indicates P < 0.001. LBMI-CT, Low BMI tumor tissue; NLBMI-CT, Non-Low BMI tumor tissue.

To explore the associations between BMI-associated intratumoral microbiota and tumor-infiltrating immune cells, we analyzed the composition of immune cells in 64 GC patients via transcriptome sequencing information and BMI data and plotted bar graphs of immune cell abundance (**Figures 5A, B**) to discover the unique features of the TIME of GC patients with different BMIs.

Correlation analysis revealed that in the NLBMI group, *g_Lachnoanaerobaculum* showed a significant positive correlation with T cell follicular helper, while Mast cells resting exhibited a significant negative correlation. *g_Stomatobaculum* demonstrated a significant positive correlation with T cell follicular helper, whereas T cells CD4 memory resting showed a significant negative correlation. In the LBMI group, *g_Enterobacter* displayed a significant positive correlation with B cells naive, while Dendritic cells activated and T cells CD4 memory resting showed significant negative correlations.*g_Abiotrophia* showed a significant negative correlation with eosinophils (**Figure 5C**). The above results indicated that the BMI-related dominant intratumoral microbiota of GC patients were significantly associated with various tumor-infiltrating immune cells, suggesting that they may play a role in regulating the immune microenvironment of GC.

### High purine metabolism in LBMI tumors

**Figure 6:**
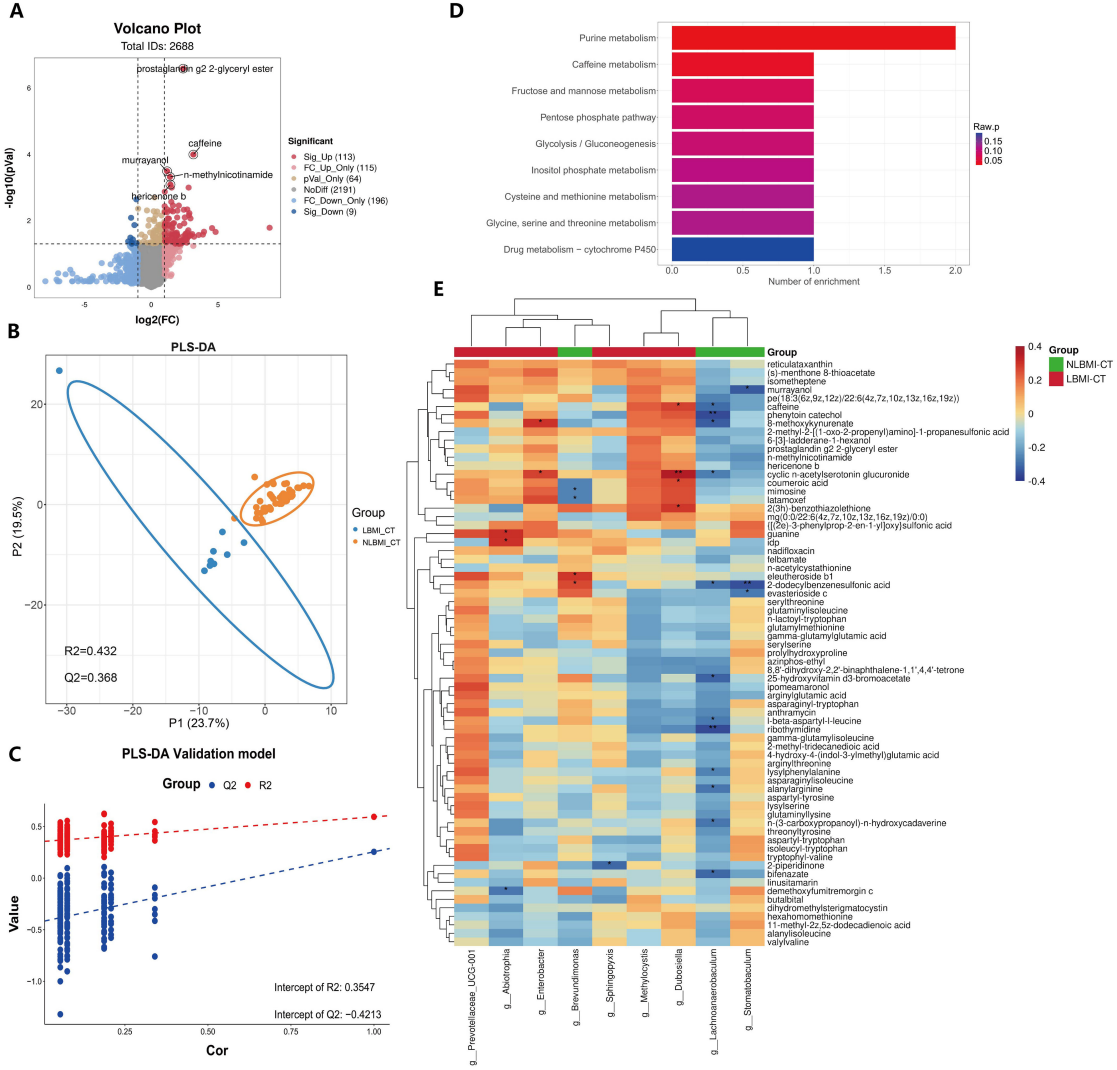
Increased purine metabolism in LBMI intratumoral environment. **(A)** Volcano plot of GC patients’ tumor tissues comparing LBM-CT and NLBMI-CT groups, with screening criteria (|log2FC|≥1, P < 0.05). **(B)** PLS-DA analysis of differential metabolites between LBM-CT and NLBMI-CT groups, with screening criteria (VIP>1, |log2FC|≥1, P < 0.05). **(C)** Validation of the PLS-DA model indicating that the model established in this study is effective. **(D)** Bar chart of enriched pathways using the KEGG database to investigate the functions of these metabolites and metabolic pathways. **(E)** Correlation heatmap showing spearman analysis between differential metabolites and genus-level intratumoral bacteria. Red indicates positive correlation; blue indicates negative correlation. The color depth represents the magnitude of the correlation coefficient, with color ranging from light to dark indicating increasing correlation value. The “*” in the figure indicates the significance level: no * indicates P value ≥ 0.05, * indicates 0.01 ≤ P < 0.05, ** indicates 0.001 ≤ P < 0.01, *** indicates P < 0.001. LBMI-CT, Low BMI tumor tissue; NLBMI-CT, Non-Low BMI tumor tissue.

Untargeted metabolomic analysis was performed on 57 tumor tissues in the transcriptome(**Supplementary Table S5**), and a total of 2688 metabolites were identified, of which 122 metabolites were significantly different between the LBMI and NLBMI groups (P<0.05,FC ≥2 or FC ≤0.5) (**Figure 6A**), and the PLS-DA scoring plot revealed that the different metabolites in the LBMI versus NLBMI tumors could be classified into two different clusters (R2Y = 0.432,Q2Y = 0.368) (**Figure 6B**). Tests of the PLS-DA model revealed that R2 > Q2 and the Q2 regression line had a negative intercept (R2 = [0.0, 0.354],Q2 = [0.0, -0.421]) (**Figure 6C**). The heatmap revealed that compared to the NLBMI group, the LBMI group had higher abundance of intratumoral purine metabolites, such as idp (**Supplementary Figure S6**).Differentially abundant metabolite KEGG enrichment analysis revealed that the LBMI group was enriched mainly in pathways such as purine metabolism and the caffeine metabolism pathway(**Figure 6D**). Genus-level differential dominant bacteria and differentially abundant metabolite correlation analysis, as shown in **Figure 6E**, revealed that the differential dominant bacteria in the NLBMI group, *g_Lachnoanaerobaculum*, were significantly negatively correlated with 12 differentially abundant metabolites, such as 8-methoxykynurenate;*g_Stomatobaculum* was significantly negatively correlated; *g_Brevundimonas* was significantly positively correlated with eleutheroside b1 and 2-dodecylbenzenesulfonic acid and significantly negatively correlated with mimosine and latamoxef. *g_Abiotrophia* in the LBMI group presented a significant negative correlation with demethoxyfumitremorgin c, whereas it presented a significant positive correlation with guanine and idp; *g_Dubosiella* presented a significant positive correlation with caffeine and four others; *g_Enterobacter* presented a significant positive correlation with cyclic n-acetylserotonin glucuronide and 8-methoxykynurenate presented a significant positive correlation; *g_Sphingopyxis* showed significant negative correlation with 2-piperidinone;*g_Prevotellaceae_UCG-001* and *g_Methylocystis* demonstrated a significant negative correlation with differentially abundant metabolites that were not significantly correlated(see **Additional File S2**). The above results revealed significant correlations between the two groups of intratumoral microbiota and metabolites, suggesting that they may further affect the biological process of gastric cancer by influencing metabolites.

## Discussion

In recent years, the relationship between GC and BMI has been studied with varying results (Schooling et al., 2015; Feng et al., 2018; Ma et al., 2021; Zhao et al., 2021). The long-term prognosis of patients with different BMIs remains unclear. Therefore, the present study was conducted to investigate BMI and GC in a large cohort. In this study, LBMI was found to be an independent risk predictor of poor prognosis, and when PSM was used to adjust for confounders and K−M survival curve analysis, it was observed that the LBMI group had a worse long-term prognosis in all patients than did the NLBMI group. This result is consistent with the findings of Feng et al (Feng et al., 2018). Several other studies have concluded that patients with LBMI have a poor prognosis (Indini et al., 2021; Ma et al., 2021; Spyrou et al., 2021). When specific subgroups, such as stage I versus stage IV patients, were analyzed, there was no significant difference in prognosis between the two groups. In contrary in stage II and III patients, LBMI patients had a significantly worse prognosis than NLBMI patients did. This finding is consistent with those of Spyrou et al. (2021) and may be because BMI has little effect on long-term prognosis in stage I versus stage IV patients. Moreover, among patients receiving postoperative adjuvant therapy, LBMI patients had worse overall survival rates and fewer benefits than NLBMI patients did, possibly because preoperative cancer-related malignancies are almost always associated with some degree of weight loss, which makes patients intolerant of postoperative adjuvant therapy side effects (Indini et al., 2021; Ma et al., 2021). Therefore, special perioperative nutritional support therapy and meticulous follow-up treatment for this special population with LBMI may improve the clinical outcome of patients.

Intratumoral microbiota are microorganisms present in tumor tissues and are now considered important regulators of many tumors, especially those of the gastrointestinal tract (Galeano Niño et al., 2022). Two recent studies have shown heterogeneity among microorganisms at different BMI states (Huang et al., 2024; Li et al., 2024). In the present study, we found significant differences in the alpha and beta diversity of the microbiota between tumor tissue and peritumoral tissue in the two groups, whereas there were no differences between intratumoral microbiota (**Figures 2A-C**). A 16S rRNA study evaluating the differences in gastric flora between 229 tumor tissues and 247 peritumoral tissues revealed that the Shannon and Simpson indices of the alpha and beta diversity of gastric intratumoral microbiota in patients with GC were significantly greater than those in paraneoplastic tissues, which is in line with the results of the present study (Liu et al., 2019). In addition, Huang et al (Li et al., 2024), There was no difference in the alpha and beta diversity of intratumoral flora between the two groups, which was consistent with the results of this study. Moreover analysis (**Figures 3A, B**) revealed a greater abundance of the differentially dominant bacterium *g_Abiotrophia* in LBMI than in NLBMI.*g_Abiotrophia* is a nutrient-variant Streptococcus species that is most commonly found in the oral cavity, frequently observed in nutritionally deficient states, and results in infective endocarditis (Mosca et al., 2021). *g_Abiotrophia* can promote fibronectin-mediated adhesion of HUVECs via DnaK and induce a proinflammatory response, leading to infective endocarditis in patients (Sasaki et al., 2021). Two studies have shown that this bacterium is highly abundant in patients with oral cancer (Mäkinen et al., 2023) and gastric cancer (Wu et al., 2018) and promotes tumor development and metastasis.

To further explore the intratumoral transcriptomic differences between the different BMI groups, a gene correlation analysis (**Figure 4E**) was performed, and the present findings revealed a significant negative correlation between *g_Abiotrophia* and P2RY12. P2RY12 was initially identified on platelets and plays an important role in platelet activation, which is also important in inflammation through the regulation of the innate and adaptive immune response (Ferrari et al., 2020). Indeed, following ADP-induced activation of P2RY12, platelets release mediators from their granules, including a variety of cytokines and chemokines, which recruit and activate leukocytes (Gómez Morillas et al., 2021). Widespread expression is also now present in many immune cells (Li et al., 2021) and it has been shown that activation of this P2RY12 receptor on dendritic cells promotes specific T- cell activation by increasing antigen endocytosis (Ben Addi et al., 2010), whereas P2RY12 inhibition induces immunosuppressive effects by decreasing antigen uptake (Mansour et al., 2020). Several recent studies have demonstrated that P2RY12 is a favorable factor for long-term prognosis in brain gliomas (Noorani et al., 2023), lung cancer (Yu et al., 2021), and hepatocellular carcinoma (Ma et al., 2022). In conclusion, we hypothesize that the inhibition of P2RY12 expression by *g_Abiotrophia* leads to the development of immunosuppression in GC patients, which leads to a poor prognosis in patients with LBMI.

As an important influence on the TIME, intratumoral microbiota can also play important roles in tumor development and metastasis by influencing immune cells (Zhou et al., 2023). In this study, *g_Abiotrophia* in the LBMI group was negatively correlated with eosinophils (**Figure 5C**). Eosinophils were first identified in peripheral blood, and it is commonly believed that eosinophils and their mediators are usually associated with deleterious effects in allergic diseases but can also induce a protective host immune response against microbial pathogens (Yousefi et al., 2008). Interestingly, a review reported that eosinophils have a beneficial effect on probiotics and may respond to local immunity by modulating homeostasis between pro- and anti-inflammatory effects (Rosenberg et al., 2016). Many studies have investigated the role of eosinophils in tumor growth control, and a review (Varricchi et al., 2017) summarizing these studies reported that the presence of eosinophils at the tumor site or in the peripheral blood is a favorable prognostic factor for most cancers. Although there is evidence that eosinophils are tumorigenic, this review demonstrated that eosinophils have an antitumor effect on patients with gastric cancer with a better prognosis via the GEO database. Two reports also reached the same conclusion (Iwasaki et al., 1986; Cuschieri et al., 2002). In addition, eosinophils can act as nonspecialized antigen-presenting cells (APCs), and upon activation by certain cytokines or other inflammatory stimuli, eosinophils can upregulate MHC class II or costimulatory markers and stimulate an initiated CD4+T-cell response *in vitro* and *in vivo* (Farhan et al., 2016). These findings suggest that eosinophils may act as helper cells in cancer and play an antitumor role. Taken together, these findings indicate that *g_Abiotrophia* may lead to tumor development and metastasis by affecting eosinophils, thus contributing to the poor prognosis of patients with LBMI gastric cancer.

Intratumoral microbiota can modulate tumor cell function by producing specific metabolites such as polyamines and short-chain fatty acids (SCFAs) (Natarajan and Pluznick, 2014). In this study, we found that LBMI-CT purine metabolism was enriched (**Figure 6D**) and that *g_Abiotrophia* was positively correlated with guanine and idp (**Figure 6E**). Purine nucleotides, such as RNA and DNA, are critical for synthesis, signaling, metabolism and energy homeostasis (Xie et al., 2024). Nutrients are required for the proliferation and differentiation of tumor cells, and guanine and idp are purine metabolites that can be synthesized into purine nucleotides through the purine metabolic pathway, which further provides nutrients for the proliferation and differentiation of tumor tissues and their development and metastasis (Yin et al., 2018). Two recent studies reported elevated nucleoside levels in GC tumor tissues (Kaji et al., 2020; Dai et al., 2021). One study, Kaji et al. (2020) reported that nucleoside concentrations were higher in GC patients with peritoneal recurrence than in GC patients without peritoneal recurrence. It is possible that increased levels of nucleosides, especially adenosine, lead to shorter survival in gastric cancer patients. Notably, a recent study (Tran et al., 2024) reported that feeding nucleosides to mice accelerated tumor growth, whereas inhibition of purine remediation slowed tumor progression, revealing a critical role of the purine remediation pathway in tumor metabolism. Interestingly, this study revealed that *g_Abiotrophia* was negatively correlated with P2RY12 (**Figure 4E**). The P2RY12 gene expresses a receptor that is a purinergic receptor and the gene is coupled to a Gi protein, resulting in reduced cAMP production (Borea et al., 2018). A recent study reported that decreased expression of P2RY12 resulted in decreased ligand production and increased cAMP production, which further led to increased synthesis of purine nucleotides or other purine metabolites within tumor tissues, thereby providing energy for tumor growth and development and promoting tumor development (Burnstock and Di Virgilio, 2013). Therefore, *g_Abiotrophia* may provide nutrients to tumor tissues by affecting P2RY12, which in turn affects on the conversion of guanine and IDP to purine nucleotides through the purine metabolic pathway.

There are several limitations to this study. First, weight loss is a common symptom in patients with GC, leading to significant differences in the distribution of BMI compared with healthy controls. This difference introduces a potential source of analytic inaccuracy and is unavoidable given the high degree of heterogeneity among GC patients. Second, the limited sample size of this study and the fact that it was a single-center retrospective analysis and that some of the missing data were not included in this study may have resulted in some selection bias. Third, compared with macrogenome sequencing, 16S rRNA gene sequencing was unable to annotate certain species at the species level, and the depth of species identification by 16S rRNA gene sequencing was relatively shallow. Lastly, basic experimental validation was not performed to draw relevant conclusions from the analysis of the histological data. Therefore, to overcome these limitations, further data validation of large-scale and prospective multicenter studies, which are combined with basic experimental validation, are needed to further validate the findings. The aforementioned limitations also offer valuable insights for future research aimed at enhancing treatment strategies for these patients.

## Conclusion

LBMI is an important independent risk factor for poor prognosis and possible immunosuppression or intolerance to postoperative adjuvant chemotherapy. *g_Abiotrophia*, a high-abundance dominant bacterium in LBMI with a negative correlation between LBMI and eosinophils, may inhibit P2RY12 to promote purine metabolism, modulate the TIME and thus contribute to gastric cancer development. Further validation in a separate cohort may be needed.

## Data Availability

The raw reads of 16S rDNA and the raw data of transcriptomewere deposited into the NCBI SRA database (Accession Number: Bioproject PRJNA1061213). The raw data of metabolome deposited into the MetaboLights database (Accession Number: MTBLS9211). The sample groupings used for the microbiome, transcriptome, and metabolome in this study are detailed in **Supplementary File 3**. The code used in this study and all supporting data are available upon request.

## Supplementary material

Supplementary Figure 1 Bioinformatics Pipeline Flowchart. This flowchart illustrates the bioinformatics pipeline used in the study, detailing the steps involved in data collection, processing, and analysis for the gastric cancer patient cohorts. Each step is visually represented to show the flow of data from initial patient selection through various analyses, including microbiota assessment, transcriptome analysis, and metabolomics evaluation. The figure provides an overview of how the different aspects of the research are integrated to assess the impact of BMI on gastric cancer outcomes and microbiota interactions.

Supplementary Figure 2 Correlation Analysis of Gastric Microbiota Samples in LBMI and NLBMI Groups and Rarefaction Curve Analysis. **(A)** Venn diagram representing the distribution of gastric microbiota and showing intersections across multiple microhabitats. **(B)** Shannon diversity measurement. **(C)** Simpson diversity measurement. **(D)** Correlation heatmap of the differential dominant genera at the genus level in tumor tissues of LBMI and NLBMI groups. The x-axis and y-axis represent bacteria. Red indicates a positive correlation, while blue indicates a negative correlation. The depth of color represents the magnitude of the Spearman correlation coefficient, with lighter colors indicating smaller values and darker colors indicating larger values. The “*” in the figure indicates the significance of the p-value: no * indicates p-value ≥ 0.05, * indicates 0.01 ≤ p < 0.05, ** indicates 0.001 ≤ p < 0.01, *** indicates p < 0.001.LBMI-CT, Low BMI tumor tissue; LBMI-NT, Low BMI adjacent normal tissue; NLBMI-CT, Non-low BMI tumor tissue; NLBMI-NT, Non-low BMI adjacent normal tissue.

Supplementary Figure 3 Differential Analysis of Microbiota in Different Tissue Sites of GC Patients in the LBMI Group. **(A)** Positive and negative bar graphs of Lefse analysis of gastric cancer microbiota in tumor and adjacent tissues of the LBMI group. The characteristic selection criterion is LDA score > 2.0. The color of the bar represents the group, and the length of the bar represents the LDA score. The LDA score indicates the influence level of the microbiota. **(B)** Evolutionary branch diagram of Lefse analysis of gastric cancer microbiota in tumor and adjacent tissues of the LBMI group. The node size represents the abundance of the species, proportional to their abundance. The color of the nodes indicates the group, with yellow nodes representing species with no significant difference between groups. Red nodes represent species significantly more abundant in the LBMI-CT group, while green nodes represent species significantly more abundant in the LBMI-NT group. The nodes within each layer, from inside to outside, represent phylum/class/order/family/genus/species, with the species annotations in each layer marking from outside to inside as phylum/class/order/family/genus/species. LBMI-CT, Low BMI tumor tissue; LBMI-NT, Low BMI adjacent tissue.

Supplementary Figure 4 Differential Analysis of Microbiota in Different Tissue Sites of GC Patients in the NLBMI Group. (A) Positive and negative bar graphs of Lefse analysis of gastric cancer microbiota in tumor and adjacent tissues of the NLBMI group. The characteristic selection criterion is LDA score > 2.0. The color of the bar represents the group, and the length of the bar represents the LDA score. The LDA score indicates the influence level of the microbiota. **(B)** Evolutionary branch diagram of Lefse analysis of gastric cancer microbiota in tumor and adjacent tissues of the NLBMI group. The node size represents the abundance of the species, proportional to their abundance. The color of the nodes indicates the group, with yellow nodes representing species with no significant difference between groups. Red nodes represent species significantly more abundant in the NLBMI-CT group, while green nodes represent species significantly more abundant in the NLBMI-NT group. The nodes within each layer, from inside to outside, represent phylum/class/order/family/genus/species, with the species annotations in each layer marking from outside to inside as phylum/class/order/family/genus/species. NLBMI-CT, Non-Low BMI tumor tissue; NLBMI-NT, Non-Low BMI adjacent tissue.

Supplementary Figure 5 Evolutionary Branch Diagram of Lefse Differential Analysis of Intratumoral Microbiota in LBMI and NLBMI Groups of GC Patients. The size of the nodes represents the abundance of the species, proportional to their abundance. The color of the nodes indicates the group, with yellow nodes representing species with no significant difference between groups. Red nodes represent species significantly more abundant in the NLBMI-CT group, while green nodes represent species significantly more abundant in the NLBMI-NT group. The nodes within each layer, from inside to outside, represent phylum/class/order/family/genus/species, with the species annotations in each layer marking from outside to inside as phylum/class/order/family/genus/species. LBMI-CT, Low BMI tumor tissue; NLBMI-CT, Non-Low BMI tumor tissue.

Supplementary Figure 6 Heatmap showing the correlation between metabolites and BMI. The heatmap displays the differential abundance of metabolites between LBMI-CT (n = 37) and NLBMI-CT (n = 16). It illustrates the relative abundance (Log) of 69 metabolites. Red indicates positive correlation; blue indicates negative correlation. The depth of the color represents the magnitude of abundance, with color ranging from light to dark indicating increasing abundance value. LBMI-CT, Low BMI tumor tissue; NLBMI-CT, Non-Low BMI tumor tissue.
