# Supplementary figures and images for: Multiomics insights into BMI-related intratumoral microbiota in gastric cancer
Source: Front Cell Infect Microbiol. 2025 Feb 18;15:1511900. doi: 10.3389/fcimb.2025.1511900 (PMC11876552; doi:10.3389/fcimb.2025.1511900)

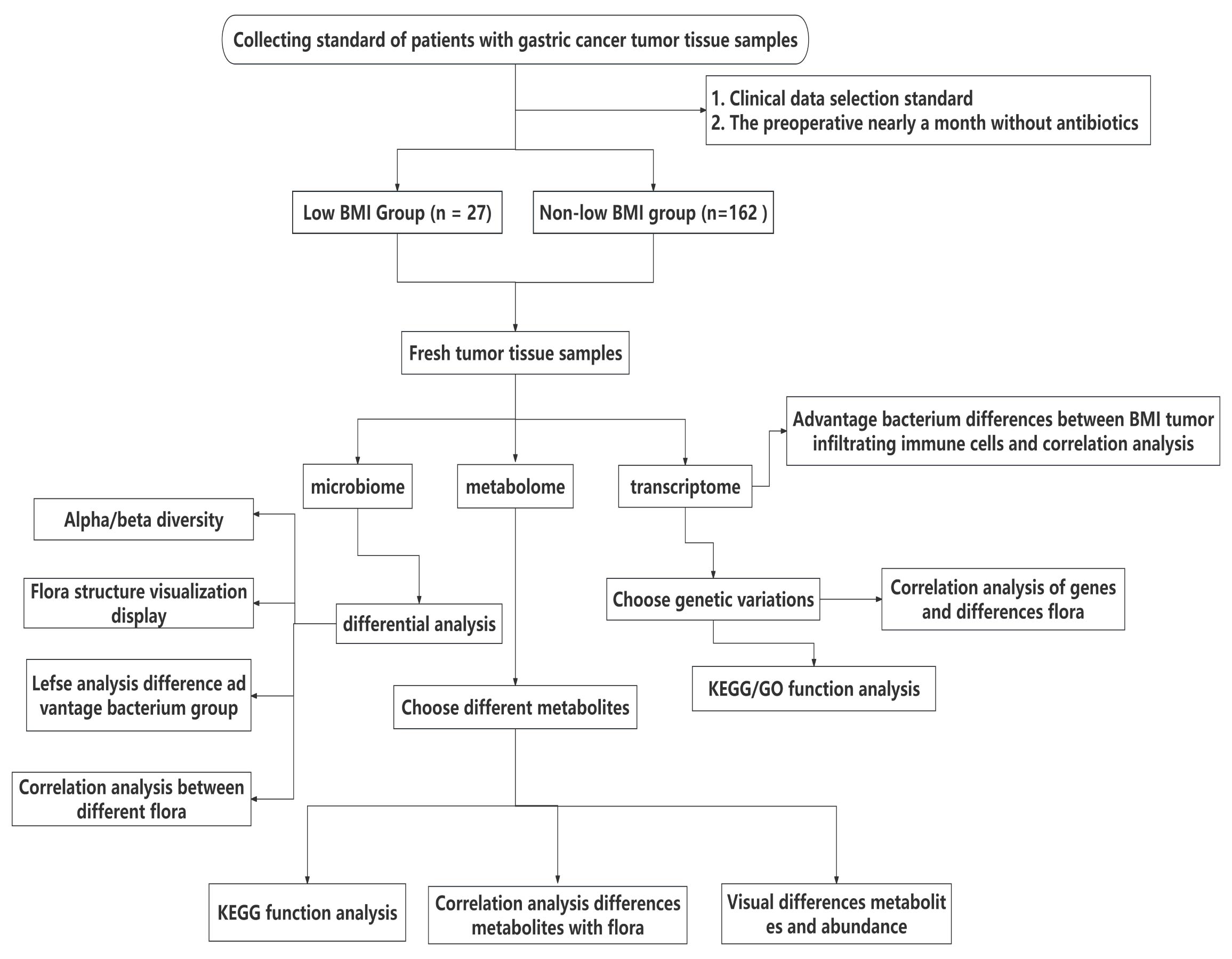

Supplement: Supplementary Figure 1 — Bioinformatics Pipeline Flowchart. This flowchart illustrates the bioinformatics pipeline used in the study, detailing the steps involved in data collection, processing, and analysis for the gastric cancer patient cohorts. Each step is visually represented to show the flow of data from initial patient selection through various analyses, including microbiota assessment, transcriptome analysis, and metabolomics evaluation. The figure provides an overview of how the different aspects of the research are integrated to assess the impact of BMI on gastric cancer outcomes and microbiota interactions. [file Image1.jpeg]

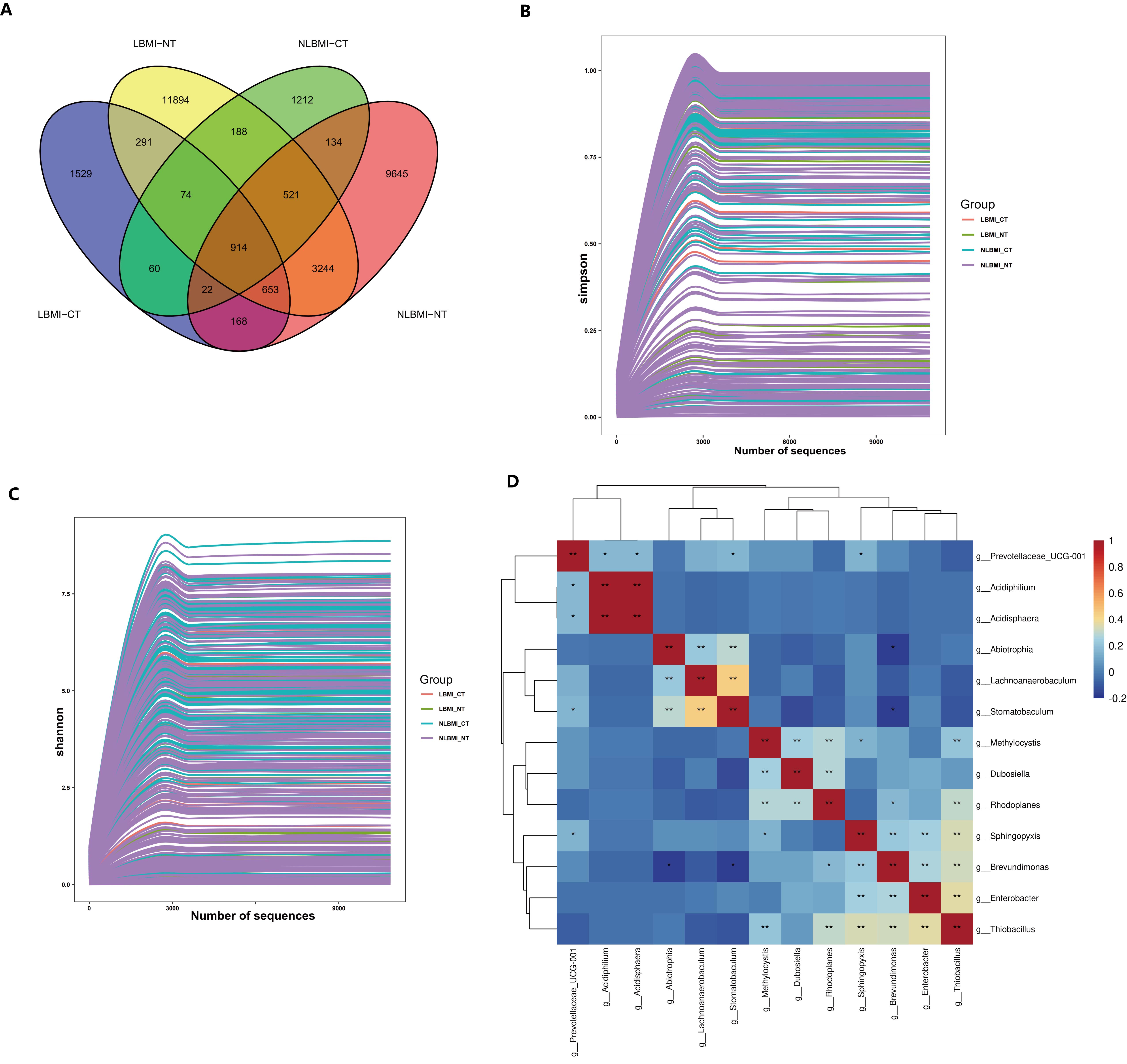

Supplement: Supplementary Figure 2 — Correlation Analysis of Gastric Microbiota Samples in LBMI and NLBMI Groups and Rarefaction Curve Analysis. (A) Venn diagram representing the distribution of gastric microbiota and showing intersections across multiple microhabitats. (B) Shannon diversity measurement. (C) Simpson diversity measurement. (D) Correlation heatmap of the differential dominant genera at the genus level in tumor tissues of LBMI and NLBMI groups. The x-axis and y-axis represent bacteria. Red indicates a positive correlation, while blue indicates a negative correlation. The depth of color represents the magnitude of the Spearman correlation coefficient, with lighter colors indicating smaller values and darker colors indicating larger values. The “*” in the figure indicates the significance of the p-value: no * indicates p-value ≥ 0.05, * indicates 0.01 ≤ p < 0.05, ** indicates 0.001 ≤ p < 0.01, *** indicates p < 0.001.LBMI-CT, Low BMI tumor tissue; LBMI-NT, Low BMI adjacent normal tissue; NLBMI-CT, Non-low BMI tumor tissue; NLBMI-NT, Non-low BMI adjacent normal tissue. [file Image2.jpeg]

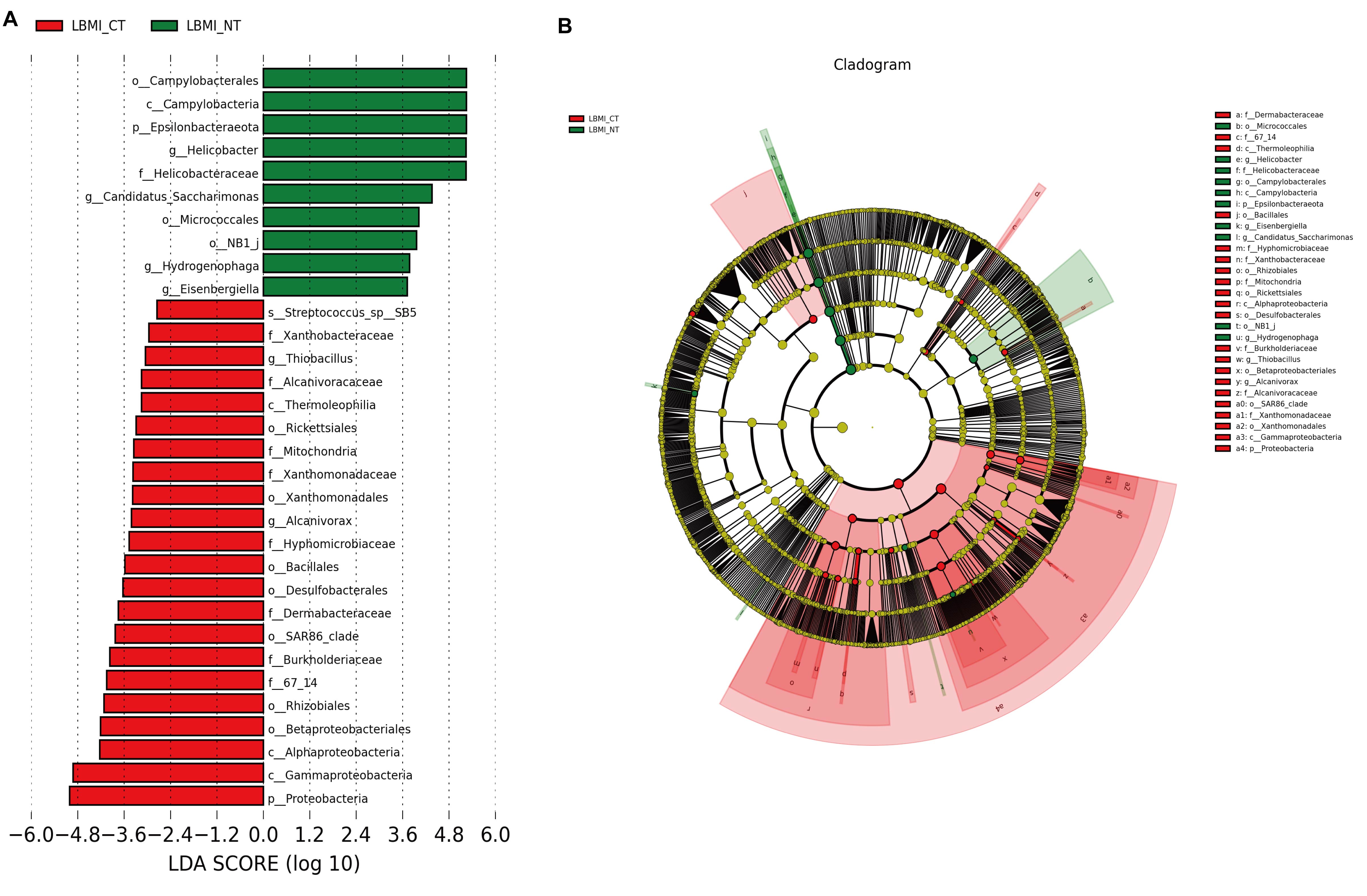

Supplement: Supplementary Figure 3 — Differential Analysis of Microbiota in Different Tissue Sites of GC Patients in the LBMI Group. (A) Positive and negative bar graphs of Lefse analysis of gastric cancer microbiota in tumor and adjacent tissues of the LBMI group. The characteristic selection criterion is LDA score > 2.0. The color of the bar represents the group, and the length of the bar represents the LDA score. The LDA score indicates the influence level of the microbiota. (B) Evolutionary branch diagram of Lefse analysis of gastric cancer microbiota in tumor and adjacent tissues of the LBMI group. The node size represents the abundance of the species, proportional to their abundance. The color of the nodes indicates the group, with yellow nodes representing species with no significant difference between groups. Red nodes represent species significantly more abundant in the LBMI-CT group, while green nodes represent species significantly more abundant in the LBMI-NT group. The nodes within each layer, from inside to outside, represent phylum/class/order/family/genus/species, with the species annotations in each layer marking from outside to inside as phylum/class/order/family/genus/species. LBMI-CT, Low BMI tumor tissue; LBMI-NT, Low BMI adjacent tissue. [file Image3.jpeg]

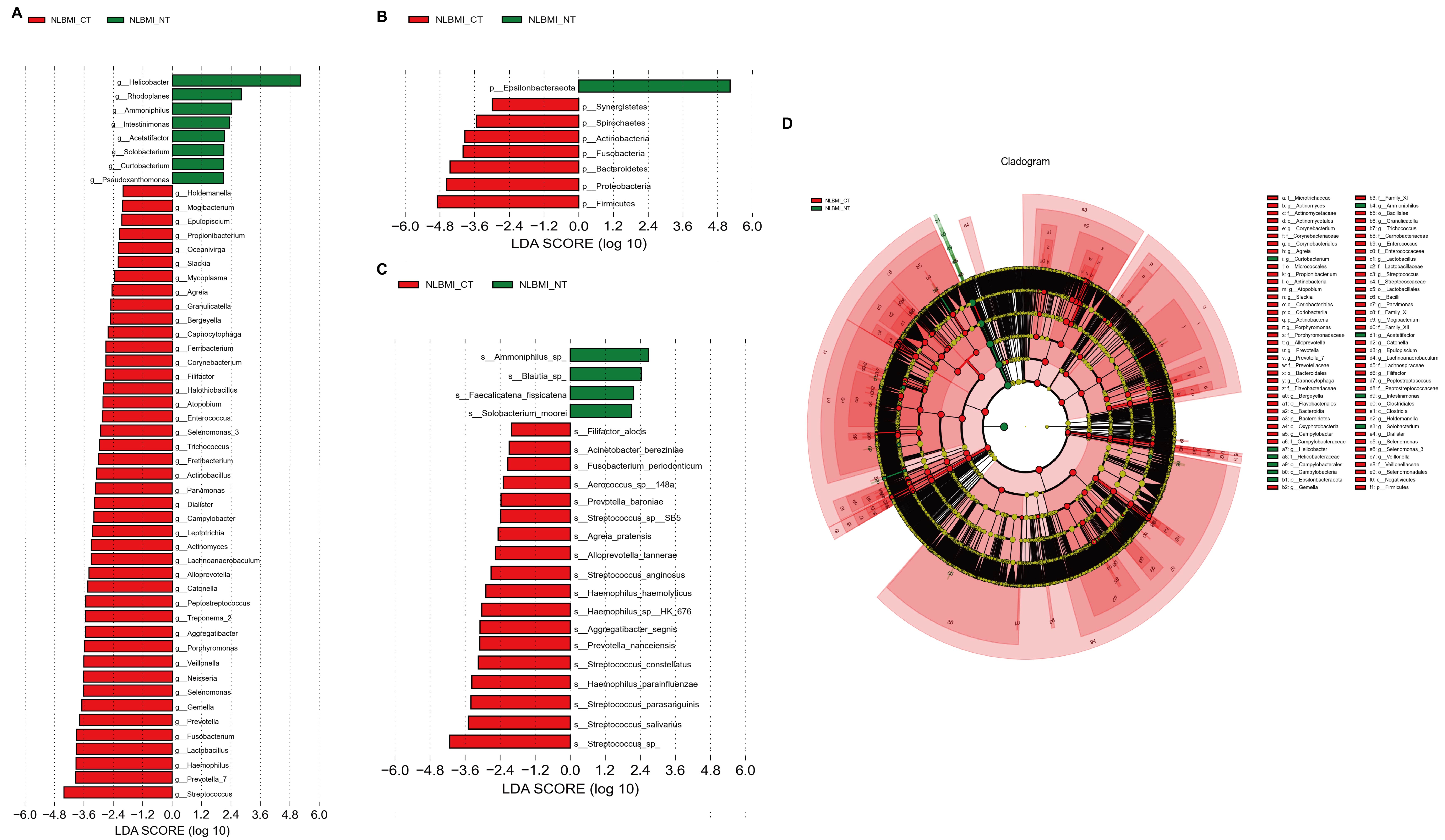

Supplement: Supplementary Figure 4 — Differential Analysis of Microbiota in Different Tissue Sites of GC Patients in the NLBMI Group. (A) Positive and negative bar graphs of Lefse analysis of gastric cancer microbiota in tumor and adjacent tissues of the NLBMI group. The characteristic selection criterion is LDA score > 2.0. The color of the bar represents the group, and the length of the bar represents the LDA score. The LDA score indicates the influence level of the microbiota. (B) Evolutionary branch diagram of Lefse analysis of gastric cancer microbiota in tumor and adjacent tissues of the NLBMI group. The node size represents the abundance of the species, proportional to their abundance. The color of the nodes indicates the group, with yellow nodes representing species with no significant difference between groups. Red nodes represent species significantly more abundant in the NLBMI-CT group, while green nodes represent species significantly more abundant in the NLBMI-NT group. The nodes within each layer, from inside to outside, represent phylum/class/order/family/genus/species, with the species annotations in each layer marking from outside to inside as phylum/class/order/family/genus/species. NLBMI-CT, Non-Low BMI tumor tissue; NLBMI-NT, Non-Low BMI adjacent tissue. [file Image4.jpeg]

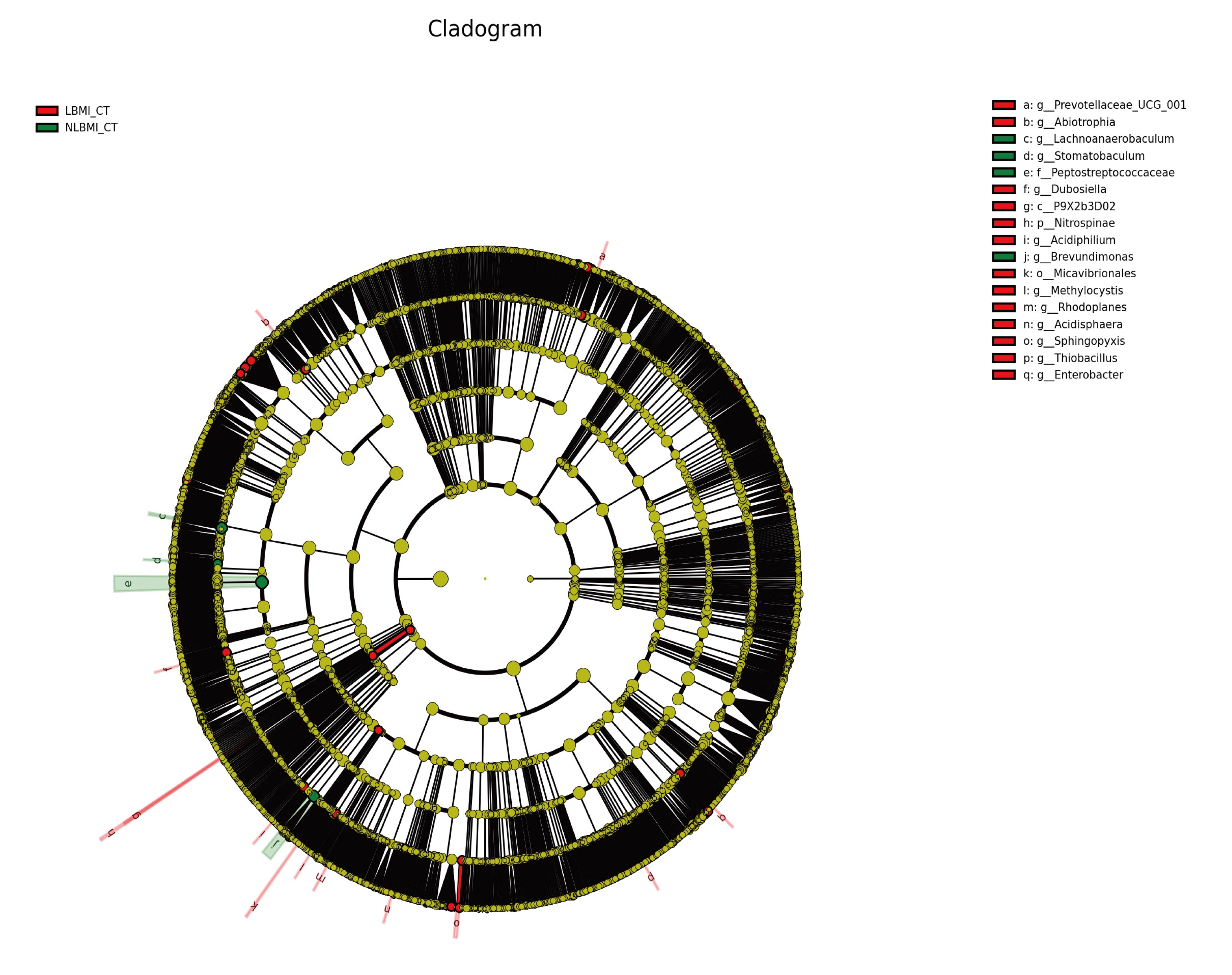

Supplement: Supplementary Figure 5 — Evolutionary Branch Diagram of Lefse Differential Analysis of Intratumoral Microbiota in LBMI and NLBMI Groups of GC Patients. The size of the nodes represents the abundance of the species, proportional to their abundance. The color of the nodes indicates the group, with yellow nodes representing species with no significant difference between groups. Red nodes represent species significantly more abundant in the NLBMI-CT group, while green nodes represent species significantly more abundant in the NLBMI-NT group. The nodes within each layer, from inside to outside, represent phylum/class/order/family/genus/species, with the species annotations in each layer marking from outside to inside as phylum/class/order/family/genus/species. LBMI-CT, Low BMI tumor tissue; NLBMI-CT, Non-Low BMI tumor tissue. [file Image5.jpeg]

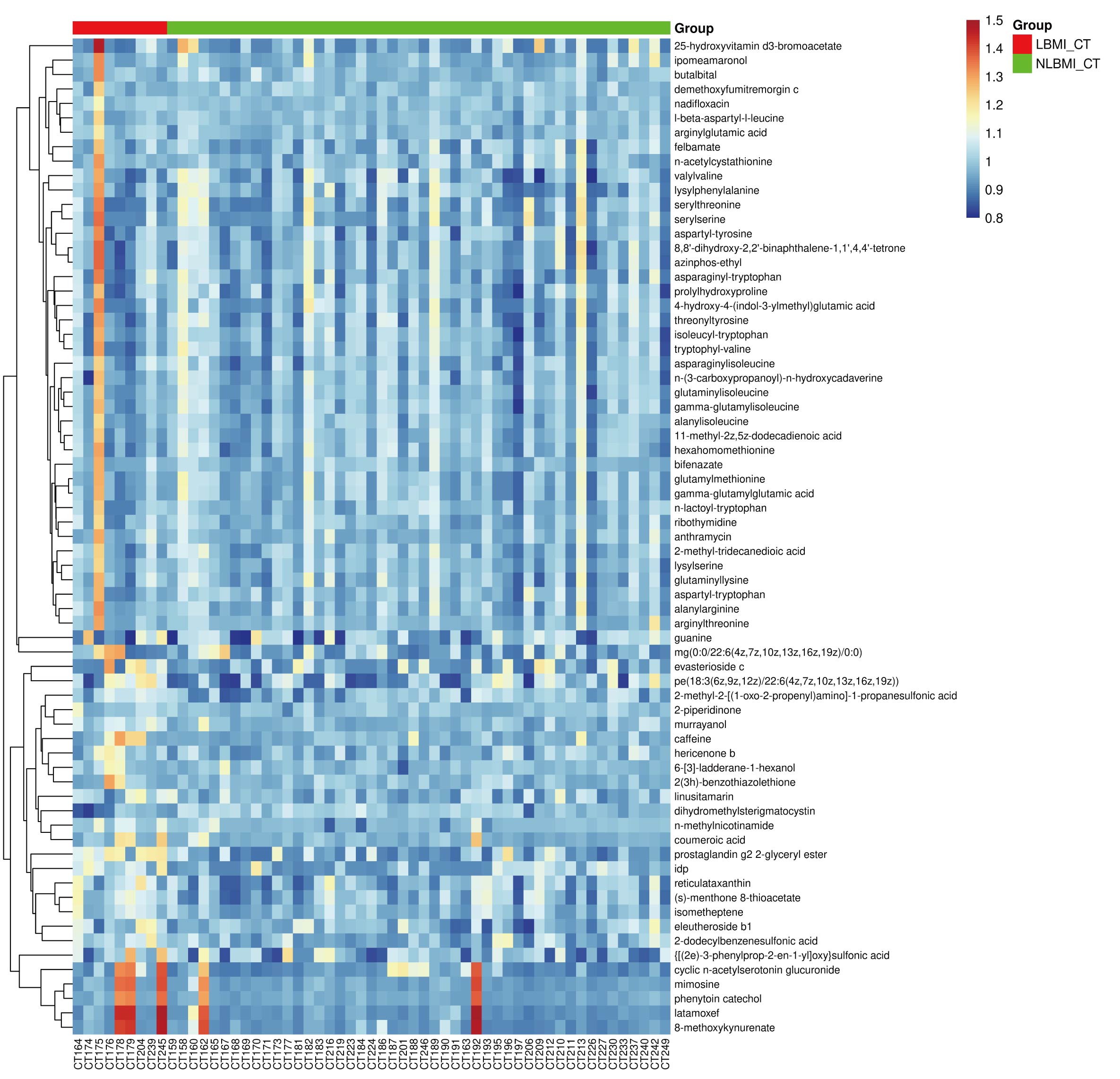

Supplement: Supplementary Figure 6 — Heatmap showing the correlation between metabolites and BMI. The heatmap displays the differential abundance of metabolites between LBMI-CT (n = 37) and NLBMI-CT (n = 16). It illustrates the relative abundance (Log) of 69 metabolites. Red indicates positive correlation; blue indicates negative correlation. The depth of the color represents the magnitude of abundance, with color ranging from light to dark indicating increasing abundance value. LBMI-CT, Low BMI tumor tissue; NLBMI-CT, Non-Low BMI tumor tissue. [file Image6.jpeg]
