# Supplementary material for: Multiomics insights into BMI-related intratumoral microbiota in gastric cancer
Source: Front Cell Infect Microbiol. 2025 Feb 18;15:1511900. doi: 10.3389/fcimb.2025.1511900 (PMC11876552; doi:10.3389/fcimb.2025.1511900)
Supplement: Supplementary file 10 [file Table4.docx]

Table S1 Clinicopathological characteristics of gastric cancer patients with and without low BMI in clinical cohort

| Variable | BMI＜18.5  (n=579) | BMI≥18.5  （n=4988） | P value |
| --- | --- | --- | --- |
| Gender |  |  | <0.001 |
| Female | 212 (36.61) | 1398 (28.03) |  |
| Male | 367 (63.39) | 3590 (71.97) |  |
| Age |  |  | <0.001 |
| ＜60 | 183 (31.61) | 2146 (43.02) |  |
| ≥60 | 396 (68.39) | 2842 (56.98) |  |
| Family history |  |  | 0.032 |
| No | 397 (68.57) | 3195 (64.05) |  |
| Yes | 182 (31.43) | 1793 (35.95) |  |
| Smoking history |  |  | 0.585 |
| No | 340 (58.72) | 2870 (57.54) |  |
| Yes | 239 (41.28) | 2118 (42.46) |  |
| Drinking history |  |  | 0.141 |
| No | 422 (72.88) | 3488 (69.93) |  |
| Yes | 157 (27.12) | 1500 (30.07) |  |
| Surgery methods |  |  | 0.019 |
| Open | 499 (86.18) | 4104 (82.28) |  |
| Laparoscopy | 80 (13.82) | 884 (17.72) |  |
| Range of resection |  |  | 0.436 |
| PG | 22 (3.80) | 144 (2.89) |  |
| DG | 327 (56.48) | 2800 (56.13) |  |
| TG | 230 (39.72) | 2044 (40.98) |  |
| Tumor location |  |  | 0.036 |
| Upper1/3 | 117 (20.21) | 1181 (23.68) |  |
| Middle1/3 | 87 (15.03) | 776 (15.56) |  |
| Lower1/3 | 352 (60.79) | 2913 (58.40) |  |
| Total | 23 (3.97) | 118 (2.37) |  |
| Differentiation |  |  | 0.020 |
| Poorly | 496 (85.66) | 4034 (80.87) |  |
| Moderately | 75 (12.95) | 858 (17.20) |  |
| Well | 8 (1.38) | 96 (1.92) |  |
| Pathological type |  |  | 0.645 |
| Adenocarcinoma | 522 (90.16) | 4525 (90.74) |  |
| MGC | 14 (2.42) | 137 (2.75) |  |
| SRCC | 43 (7.43) | 325 (6.52) |  |
| Vascular tumor thrombus |  |  | 0.229 |
| No | 287 (49.57) | 2604 (52.21) |  |
| Yes | 292 (50.43) | 2384 (47.79) |  |
| Nerve invasion |  |  | 0.011 |
| No | 248 (42.83) | 2416 (48.44) |  |
| Yes | 331 (57.17) | 2572 (51.56) |  |
| Maximum tumor diameter |  |  | 0.007 |
| ＜5 | 306 (52.85) | 2930 (58.74) |  |
| ≥5 | 273 (47.15) | 2058 (41.26) |  |
| pT Satge |  |  | 0.002 |
| T1 | 106 (18.31) | 1123 (22.51) |  |
| T2 | 50 (8.64) | 605 (12.13) |  |
| T3 | 38 (6.56) | 331 (6.64) |  |
| T4 | 385 (66.49) | 2929 (58.72) |  |
| pN Stage |  |  | 0.004 |
| N0 | 156 (26.94) | 1674 (33.56) |  |
| N1 | 92 (15.89) | 836 (16.76) |  |
| N2 | 126 (21.76) | 998 (20.01) |  |
| N3 | 205 (35.41) | 1480 (29.67) |  |
| pM Stage |  |  | 0.871 |
| M0 | 564 (97.41) | 4853 (97.29) |  |
| M1 | 15 (2.59) | 135 (2.71) |  |
| pTNM Stage |  |  | <0.001 |
| I | 114 (19.69) | 1278 (25.62) |  |
| II | 97 (16.75) | 963 (19.31) |  |
| III | 353 (60.97) | 2612 (52.37) |  |
| IV | 15 (2.59) | 135 (2.71) |  |
| Postoperative adjuvant therapy |  |  | <0.001 |
| No | 378 (65.28) | 2680 (53.73) |  |
| Yes | 201 (34.72) | 2308 (46.27) |  |
| Pre-CEA |  |  | 0.105 |
| Negative | 449 (78.50) | 3979 (81.30) |  |
| Positive | 123 (21.50) | 915 (18.70) |  |
| Pre-CA199 |  |  | 0.039 |
| Negative | 426 (77.74) | 3800 (81.39) |  |
| Positive | 122 (22.26) | 869 (18.61) |  |
| Complication |  |  | <0.001 |
| No | 518 (89.46) | 4654 (93.30) |  |
| Yes | 61 (10.54) | 334 (6.70) |  |
| Recurrence |  |  | 0.618 |
| No | 507 (87.56) | 4403 (88.27) |  |
| Yes | 72 (12.44) | 585 (11.73) |  |

BMI:Body Mass Index,PG:proximal gastrectomy,DG:Distal gastrectomy,TG:total gastrectomyMGC:Mucinous adenocarcinoma,SRCC:signet-ring cell carcinoma,Pre-:Pre-operation.P < 0.05 was considered significant.
