# Supplementary material for: Multiomics insights into BMI-related intratumoral microbiota in gastric cancer
Source: Front Cell Infect Microbiol. 2025 Feb 18;15:1511900. doi: 10.3389/fcimb.2025.1511900 (PMC11876552; doi:10.3389/fcimb.2025.1511900)
Supplement: Supplementary file 11 [file Table5.docx]

TableS2 Clinicopathological features of clinical cohort after PSM

| Variable | BMI＜18.5  (n=579) | BMI≥18.5  （n= 2191） | P value |
| --- | --- | --- | --- |
| Gender |  |  | 0.186 |
| Female | 212 (36.61) | 738 (33.68) |  |
| Male | 367 (63.39) | 1453 (66.32) |  |
| Age |  |  | 0.553 |
| ＜60 | 183 (31.61) | 721 (32.91) |  |
| ≥60 | 396 (68.39) | 1470 (67.09) |  |
| Family history |  |  | 0.362 |
| No | 397 (68.57) | 1545 (70.52) |  |
| Yes | 182 (31.43) | 646 (29.48) |  |
| Surgery methods |  |  | 0.904 |
| Open | 499 (86.18) | 1884 (85.99) |  |
| Laparoscopy | 80 (13.82) | 307 (14.01) |  |
| Range of resection |  |  | 0.209 |
| PG | 22 (3.80) | 58 (2.65) |  |
| DG | 327 (56.48) | 1301 (59.38) |  |
| TG | 230 (39.72) | 832 (37.97) |  |
| Differentiation |  |  | 0.780 |
| Poorly | 496 (85.66) | 1857 (84.76) |  |
| Moderately | 75 (12.95) | 307 (14.01) |  |
| Well | 8 (1.38) | 27 (1.23) |  |
| Pathological type |  |  | 0.766 |
| Adenocarcinoma | 522 (90.16) | 1977 (90.23) |  |
| MGC | 14 (2.42) | 63 (2.88) |  |
| SRCC | 43 (7.43) | 151 (6.89) |  |
| Vascular tumor thrombus |  |  | 0.800 |
| No | 287 (49.57) | 1099 (50.16) |  |
| Yes | 292 (50.43) | 1092 (49.84) |  |
| Nerve invasion |  |  | 0.851 |
| No | 248 (42.83) | 948 (43.27) |  |
| Yes | 331 (57.17) | 1243 (56.73) |  |
| Maximum tumor diameter |  |  | 0.336 |
| ＜5 | 306 (52.85) | 1207 (55.09) |  |
| ≥5 | 273 (47.15) | 984 (44.91) |  |
| TNM Stage |  |  | 0.613 |
| I | 114 (19.69) | 461 (21.04) |  |
| II | 97 (16.75) | 393 (17.94) |  |
| III | 353 (60.97) | 1270 (57.96) |  |
| IV | 15 (2.59) | 67 (3.06) |  |
| Postoperative adjuvant therapy |  |  | 0.563 |
| No | 378 (65.28) | 1402 (63.99) |  |
| Yes | 201 (34.72) | 789 (36.01) |  |
| Pre-CEA |  |  | 0.697 |
| Negative | 449 (77.55) | 1721 (78.55) |  |
| Positive | 123 (21.24) | 437 (19.95) |  |
| Pre-CA199 |  |  | 0.739 |
| Negative | 426 (73.58) | 1640 (74.85) |  |
| Positive | 122 (21.07) | 430 (19.63) |  |

BMI:Body Mass Index,PG:proximal gastrectomy,DG:Distal gastrectomy,TG:total gastrectomyMGC:Mucinous adenocarcinoma,SRCC:signet-ring cell carcinoma,Pre-:Pre-operation.PSM:Propensity score matching method.P < 0.05 was considered significant.
