# Supplementary material for: Multiomics insights into BMI-related intratumoral microbiota in gastric cancer
Source: Front Cell Infect Microbiol. 2025 Feb 18;15:1511900. doi: 10.3389/fcimb.2025.1511900 (PMC11876552; doi:10.3389/fcimb.2025.1511900)
Supplement: Supplementary file 12 [file Table6.docx]

TableS3 Clinicopathological features of patients with gastric cancer before and after receiving postoperative adjuvant therapy for PSM

| Variable | Before PSM | | P value | After PSM | | P value |
| --- | --- | --- | --- | --- | --- | --- |
|  | BMI＜18.5  (n=579) | BMI≥18.5  （n= 2191） |  | BMI＜18.5  (n=201) | BMI≥18.5  （n= 782） |  |
| Gender |  |  | <0.001 |  |  | 0.673 |
| Female | 84 (41.79) | 623 (26.99) |  | 84 (41.79) | 314 (40.15) |  |
| Male | 117 (58.21) | 1685 (73.01) |  | 117 (58.21) | 468 (59.85) |  |
| Age |  |  | 0.121 |  |  | 0.879 |
| ＜60 | 94 (46.77) | 1211 (52.47) |  | 94 (46.77) | 361 (46.16) |  |
| ≥60 | 107 (53.23) | 1097 (47.53) |  | 107 (53.23) | 421 (53.84) |  |
| Family history |  |  | 0.092 |  |  | 0.752 |
| No | 135 (67.16) | 1411 (61.14) |  | 135 (67.16) | 516 (65.98) |  |
| Yes | 66 (32.84) | 897 (38.86) |  | 66 (32.84) | 266 (34.02) |  |
| Surgery methods |  |  | 0.631 |  |  | 0.857 |
| Open | 176 (87.56) | 1993 (86.35) |  | 176 (87.56) | 681 (87.08) |  |
| Laparoscopy | 25 (12.44) | 315 (13.65) |  | 25 (12.44) | 101 (12.92) |  |
| Range of resection |  |  | 0.544 |  |  | 0.236 |
| PG | 6 (2.99) | 56 (2.43) |  | 6 (2.99) | 11 (1.41) |  |
| DG | 113 (56.22) | 1222 (52.95) |  | 113 (56.22) | 424 (54.22) |  |
| TG | 82 (40.80) | 1030 (44.63) |  | 82 (40.80) | 347 (44.37) |  |
| Differentiation |  |  | 0.327 |  |  | 0.199 |
| Poorly | 179 (89.05) | 1966 (85.18) |  | 179 (89.05) | 674 (86.19) |  |
| Moderately | 21 (10.45) | 327 (14.17) |  | 21 (10.45) | 107 (13.68) |  |
| Well | 1 (0.50) | 15 (0.65) |  | 1 (0.50) | 1 (0.13) |  |
| Pathological type |  |  | 0.331 |  |  | 0.488 |
| Adenocarcinoma | 174 (86.57) | 2064 (89.43) |  | 174 (86.57) | 698 (89.26) |  |
| MGC | 7 (3.48) | 80 (3.47) |  | 7 (3.48) | 26 (3.32) |  |
| SRCC | 20 (9.95) | 164 (7.11) |  | 20 (9.95) | 58 (7.42) |  |
| Vascular tumor thrombus |  |  | 0.618 |  |  | 0.306 |
| No | 84 (41.79) | 923 (39.99) |  | 84 (41.79) | 296 (37.85) |  |
| Yes | 117 (58.21) | 1385 (60.01) |  | 117 (58.21) | 486 (62.15) |  |
| Nerve invasion |  |  | 0.116 |  |  | 0.764 |
| No | 60 (29.85) | 816 (35.36) |  | 60 (29.85) | 225 (28.77) |  |
| Yes | 141 (70.15) | 1492 (64.64) |  | 141 (70.15) | 557 (71.23) |  |
| Maximum tumor diameter |  |  | 0.753 |  |  | 0.673 |
| ＜5 | 100 (49.75) | 1175 (50.91) |  | 100 (49.75) | 376 (48.08) |  |
| ≥5 | 101 (50.25) | 1133 (49.09) |  | 101 (50.25) | 406 (51.92) |  |
| TNM Stage |  |  | 0.195 |  |  | 0.694 |
| I | 17 (8.46) | 163 (7.06) |  | 17 (8.46) | 54 (6.91) |  |
| II | 32 (15.92) | 481 (20.84) |  | 32 (15.92) | 130 (16.62) |  |
| III | 140 (69.65) | 1574 (68.20) |  | 140 (69.65) | 563 (71.99) |  |
| IV | 12 (5.97) | 90 (3.90) |  | 12 (5.97) | 35 (4.48) |  |
| Pre-CEA |  |  | 0.613 |  |  | 0.646 |
| Negative | 160 (79.60) | 1826 (79.12) |  | 160 (79.60) | 634 (81.07) |  |
| Positive | 40 (19.90) | 452 (19.58) |  | 40 (19.90) | 140 (17.90) |  |
| Pre-CA199 |  |  | 0.755 |  |  | 0.754 |
| Negative | 144 (71.64) | 1704 (73.83) |  | 144 (71.64) | 539 (68.93) |  |
| Positive | 44 (21.89) | 477 (20.67) |  | 44 (21.89) | 189 (24.17) |  |

BMI:Body Mass Index,PG:proximal gastrectomy,DG:Distal gastrectomy,TG:total gastrectomyMGC:Mucinous adenocarcinoma,SRCC:signet-ring cell carcinoma,Pre-:Pre-operation.PSM:Propensity score matching method.P < 0.05 was considered significant.
