# Supplementary material for: Multiomics insights into BMI-related intratumoral microbiota in gastric cancer
Source: Front Cell Infect Microbiol. 2025 Feb 18;15:1511900. doi: 10.3389/fcimb.2025.1511900 (PMC11876552; doi:10.3389/fcimb.2025.1511900)
Supplement: Supplementary file 13 [file Table7.docx]

TableS4 The clinicopathological characteristics of gastric cancer patients were analyzed by bioinformatics

| Variable | BMI＜18.5  (n=27) | BMI≥18.5  （n=162） | P value |
| --- | --- | --- | --- |
| Gender |  |  | 0.261 |
| Female | 10 (37.04) | 43 (26.54) |  |
| Male | 17 (62.96) | 119 (73.46) |  |
| Age |  |  | 0.069 |
| ＜60 | 5 (18.52) | 59 (36.42) |  |
| ≥60 | 22 (81.48) | 103 (63.58) |  |
| Family history |  |  | 0.892 |
| No | 23 (85.19) | 143 (88.27) |  |
| Yes | 4 (14.81) | 19 (11.73) |  |
| Smoking history |  |  | 0.217 |
| No | 20 (74.07) | 100 (61.73) |  |
| Yes | 7 (25.93) | 62 (38.27) |  |
| Drinking history |  |  | 0.460 |
| No | 21 (77.78) | 109 (67.28) |  |
| Yes | 6 (22.22) | 53 (32.72) |  |
| Tumor location |  |  | 0.513 |
| Gastric cardia | 5 (18.52) | 36 (22.22) |  |
| Gastric body | 5 (18.52) | 43 (26.54) |  |
| Gastric antrum | 17 (62.96) | 83 (51.23) |  |
| Differentiation |  |  | 0.170 |
| Poor | 10 (37.04) | 78 (48.15) |  |
| Moderate | 14 (51.85) | 54 (33.33) |  |
| Well | 3 (11.11) | 30 (18.52) |  |
| Pathological type |  |  | 0.377 |
| Adenocarcinoma | 25 (92.59) | 155 (95.68) |  |
| MGC | 1 (3.70) | 3 (1.85) |  |
| SRCC | 1 (3.70) | 4 (2.47) |  |
| T Satge |  |  | 0.757 |
| T1 | 3 (11.11) | 21 (12.96) |  |
| T2 | 3 (11.11) | 19 (11.73) |  |
| T3 | 6 (22.22) | 23 (14.20) |  |
| T4 | 15 (55.56) | 99 (61.11) |  |
| N Stage |  |  | 0.783 |
| N0 | 7 (25.93) | 54 (33.33) |  |
| N1 | 7 (25.93) | 30 (18.52) |  |
| N2 | 5 (18.52) | 28 (17.28) |  |
| N3 | 8 (29.63) | 50 (30.86) |  |
| M Stage |  |  | 0.234 |
| M0 | 23 (85.19) | 152 (93.83) |  |
| M1 | 4 (14.81) | 10 (6.17) |  |
| TNM Stage |  |  | 0.450 |
| I | 5 (18.52) | 32 (19.75) |  |
| II | 6 (22.22) | 35 (21.60) |  |
| III | 12 (44.44) | 85 (52.47) |  |
| IV | 4 (14.81) | 10 (6.17) |  |
| Pre-CEA |  |  | 0.677 |
| Negative | 25 (92.59) | 142 (87.65) |  |
| Positive | 2 (7.41) | 20 (12.35) |  |
| Pre-CA199 |  |  | 0.731 |
| Negative | 21 (77.78) | 121 (74.69) |  |
| Positive | 6 (22.22) | 41 (25.31) |  |

BMI:Body Mass Index,PG:proximal gastrectomy,DG:Distal gastrectomy,TG:total gastrectomyMGC:Mucinous adenocarcinoma,SRCC:signet-ring cell carcinoma,Pre-:Pre-operation.P < 0.05 was considered significant.
