# Supplementary material for: Multiomics insights into BMI-related intratumoral microbiota in gastric cancer
Source: Front Cell Infect Microbiol. 2025 Feb 18;15:1511900. doi: 10.3389/fcimb.2025.1511900 (PMC11876552; doi:10.3389/fcimb.2025.1511900)
Supplement: Supplementary file 14 [file Table8.docx]

TableS5 The clinicopathological characteristics of gastric cancer patients were analyzed by metabolomics

| Variable | BMI＜18.5  (n=27) | BMI≥18.5  （n=162） | P value |
| --- | --- | --- | --- |
| Gender |  |  | 0.482 |
| Female | 3 (33.33) | 8 (16.67) |  |
| Male | 6 (66.67) | 40 (83.33) |  |
| Age |  |  | 0.069 |
| ＜60 | 2 (22.22) | 21 (43.75) |  |
| ≥60 | 7 (77.78) | 27 (56.25) |  |
| Family history |  |  | 1.000 |
| No | 8 (88.89) | 43 (89.58) |  |
| Yes | 1 (11.11) | 5 (10.42) |  |
| Smoking history |  |  | 0.761 |
| No | 6 (66.67) | 25 (54.16) |  |
| Yes | 3 (33.33) | 22 (45.83) |  |
| Drinking history |  |  | 0.347 |
| No | 8 (88.89) | 32 (66.67) |  |
| Yes | 1 (11.11) | 16 (33.33) |  |
| Tumor location |  |  | 0.804 |
| Gastric cardia | 1 (11.11) | 11 (22.92) |  |
| Gastric body | 3 (33.33) | 15 (31.25) |  |
| Gastric antrum | 5 (55.56) | 22 (45.83) |  |
| Differentiation |  |  | 0.155 |
| Poor | 2 (22.22) | 23 (47.92) |  |
| Moderate | 6 (66.67) | 15 (31.25) |  |
| Well | 1 (11.11) | 10 (20.83) |  |
| Pathological type |  |  | 1.000 |
| Adenocarcinoma | 9 (100.00) | 45 (93.75) |  |
| MGC | 0 (0.00) | 2 (4.17) |  |
| SRCC | 0 (0.00) | 1 (2.08) |  |
| T Satge |  |  | 0.888 |
| T1 | 0 (0.00) | 2 (4.17) |  |
| T2 | 2 (22.22) | 7 (14.58) |  |
| T3 | 1 (11.11) | 5 (10.42) |  |
| T4 | 6 (66.67) | 34 (70.83) |  |
| N Stage |  |  | 0.365 |
| N0 | 3 (33.33) | 12 (25.00) |  |
| N1 | 3 (33.33) | 10 (20.83) |  |
| N2 | 2 (22.22) | 7 (14.58) |  |
| N3 | 1 (11.11) | 19 (39.58) |  |
| M Stage |  |  | 0.173 |
| M0 | 7 (77.78) | 45 (93.75) |  |
| M1 | 2 (22.22) | 3 (6.25) |  |
| TNM Stage |  |  | 0.193 |
| I | 2 (22.22) | 6 (12.50) |  |
| II | 2 (22.22) | 10 (20.83) |  |
| III | 3 (33.33) | 29 (60.42) |  |
| IV | 2 (22.22) | 3 (6.25) |  |
| Pre-CEA |  |  | 1.000 |
| Negative | 7 (77.78) | 37 (77.08) |  |
| Positive | 2 (22.22) | 11 (22.92) |  |
| Pre-CA199 |  |  | 0.347 |
| Negative | 8 (88.89) | 32 (66.67) |  |
| Positive | 1 (11.11) | 16 (33.33) |  |

BMI:Body Mass Index,PG:proximal gastrectomy,DG:Distal gastrectomy,TG:total gastrectomyMGC:Mucinous adenocarcinoma,SRCC:signet-ring cell carcinoma,Pre-:Pre-operation.P < 0.05 was considered significant.
